# *PLCG2* protective variant p.P522R modulates tau pathology and disease progression in patients with mild cognitive impairment

**DOI:** 10.1007/s00401-020-02138-6

**Published:** 2020-03-12

**Authors:** Luca Kleineidam, Vincent Chouraki, Tomasz Próchnicki, Sven J. van der Lee, Laura Madrid-Márquez, Holger Wagner-Thelen, Ilker Karaca, Leonie Weinhold, Steffen Wolfsgruber, Anne Boland, Pamela V. Martino Adami, Piotr Lewczuk, Julius Popp, Frederic Brosseron, Iris E. Jansen, Marc Hulsman, Johannes Kornhuber, Oliver Peters, Claudine Berr, Reinhard Heun, Lutz Frölich, Christophe Tzourio, Jean-François Dartigues, Michael Hüll, Ana Espinosa, Isabel Hernández, Itziar de Rojas, Adelina Orellana, Sergi Valero, Najada Stringa, Natasja M. van Schoor, Martijn Huisman, Philip Scheltens, Alzheimer’s Disease Neuroimaging Initiative (ADNI), Luca Kleineidam, Ilker Karaca, Michael T. Heneka, Wolfgang Maier, Anja Schneider, Michael Wagner, Vincent Chouraki, Phillipe Amouyel, Jean-Charles Lambert, Tomasz Próchnicki, Eicke Latz, Sven J. van der Lee, Iris E. Jansen, Marc Hulsman, Philip Scheltens, Wiesje M. van der Flier, Henne Holstege, Laura Madrid-Márquez, Antonio González-Pérez, Mª Eugenia Sáez, Holger Wagner-Thelen, Pamela V. Martino Adami, Frank Jessen, Alfredo Ramirez, Leonie Weinhold, Matthias Schmid, Steffen Wolfsgruber, Frederic Brosseron, Anne Boland, Jean-Francois Deleuze, Piotr Lewczuk, Johannes Kornhuber, Julius Popp, Oliver Peters, Claudine Berr, Reinhard Heun, Lutz Frölich, Christophe Tzourio, Jean-François Dartigues, Michael Hüll, Ana Espinosa, Isabel Hernández, Itziar de Rojas, Adelina Orellana, Sergi Valero, Agustin Ruiz, Lluis Tarraga, Merce Boada, Najada Stringa, Natasja M. van Schoor, Martijn Huisman, Eckart Rüther, Jens Wiltfang, Martin Scherer, Steffi Riedel-Heller, Eckart Rüther, Jean-Francois Deleuze, Jens Wiltfang, Lluis Tarraga, Matthias Schmid, Martin Scherer, Steffi Riedel-Heller, Michael T. Heneka, Philippe Amouyel, Frank Jessen, Merce Boada, Wolfgang Maier, Anja Schneider, Antonio González-Pérez, Wiesje M. van der Flier, Michael Wagner, Jean-Charles Lambert, Henne Holstege, Mª Eugenia Sáez, Eicke Latz, Agustin Ruiz, Alfredo Ramirez

**Affiliations:** 1 Department of Neurodegenerative Diseases and Geriatric Psychiatry, University Hospital Bonn, Bonn, Germany; 2 Division of Neurogenetics and Molecular Psychiatry, Department of Psychiatry and Psychotherapy, Medical Faculty, University of Cologne, Cologne, Germany; 3 German Center for Neurodegenerative Diseases (DZNE), Bonn, Germany; 4 Univ. Lille, Inserm, CHU Lille, Institut Pasteur de Lille, U1167-RID-AGE-Facteurs de risque Et déterminants moléculaires des maladies liées au vieillissement, Lille, France; 5 Epidemiology and Public Health Department, Centre Hospitalier Universitaire de Lille, Lille, France; 6 Institute of Innate Immunity, University Hospitals Bonn, Bonn, Germany; 7 Department of Neurology, Alzheimer Center Amsterdam, Amsterdam Neuroscience, Amsterdam UMC, Vrije Universiteit Amsterdam, Amsterdam, The Netherlands; 8 Department of Clinical Genetics, Amsterdam Neuroscience, Amsterdam UMC, Vrije Universiteit Amsterdam, Amsterdam, The Netherlands; 9 Andalusian Bioinformatics Research Centre (CAEBi), Seville, Spain; 10 Institute of Medical Biometry, Informatics and Epidemiology, University Hospital of Bonn, Bonn, Germany; 11 Centre National de Recherche en Génomique Humaine (CNRGH), Institut de Biologie François Jacob, CEA, Université Paris-Saclay, Évry, France; 12 Department of Psychiatry and Psychotherapy, Universitätsklinikum Erlangen, and Friedrich-Alexander Universität Erlangen-Nürnberg, Erlangen, Germany; 13 Department of Neurodegeneration Diagnostics, Medical University of Białystok, Białystok, Poland; 14 Department of Psychiatry, Lausanne University Hospital, Prilly, Switzerland; 15 Department of Geriatric Psychiatry, University Hospital of Psychiatry Zurich, Zurich, Switzerland; 16 Department of Psychiatry, Charité – Universitätsmedizin Berlin, Berlin, Germany; 17 INSERM, University Montpellier, Neuropsychiatry: Epidemiological and Clinical Research, Montpellier, France; 18 Department of Psychiatry and Psychotherapy, University Hospital Bonn, 53127 Bonn, Germany; 19 Department of Geriatric Psychiatry, Medical Faculty Mannheim, Central Institute of Mental Health, University of Heidelberg, Mannheim, Germany; 20 Inserm, Bordeaux Population Health Research Center, UMR1219, University of Bordeaux, Bordeaux, France; 21 Department of Psychiatry and Psychotherapy, Center for Psychiatry, Clinic for Geriatric Psychiatry and Psychotherapy Emmendingen, University of Freiburg, Freiburg, Germany; 22 Research Center and Memory Clinic, Fundació ACE, Institut Català de Neurociències Aplicades-Universitat Internacional de Catalunya-Barcelona, Barcelona, Spain; 23 Centro de Investigación Biomédica en Red sobre Enfermedades Neurodegenerativas (CIBERNED), Instituto de Salud Carlos III, Madrid, Spain; 24 Department of Epidemiology and Biostatistics, Amsterdam Public Health Research Institute, Amsterdam UMC–Vrije Universiteit Amsterdam, Amsterdam, The Netherlands; 25 Department of Psychiatry and Psychotherapy, University Medical Center Göttingen, Göttingen, Germany; 26 German Center for Neurodegenerative Diseases (DZNE), Göttingen, Germany; 27 iBiMED, Medical Sciences Department, University of Aveiro, Aveiro, Portugal; 28 Department of Primary Medical Care, University Medical Center Hamburg-Eppendorf, Hamburg, Germany; 29 Institute of Social Medicine, Occupational Health and Public Health, University of Leipzig, Leipzig, Germany; 30 Division of Infectious Diseases and Immunology, Department of Medicine, University of Massachusetts Medical School, Worcester, MA USA; 31 Department of Psychiatry and Psychotherapy, Medical Faculty, University of Cologne, Cologne, Germany; 32 Centre for Molecular Inflammation Research (CEMIR), Norwegian University of Science and Technology, Trondheim, Norway; 33 Department of Biochemical Diagnostics, University Hospital of Białystok, Białystok, Poland; 34 Department of Complex Trait Genetics, Center for Neurogenomics and Cognitive Research, Amsterdam Neuroscience, Vrije Universiteit Amsterdam, Amsterdam, The Netherlands; 35 DZNE, German Center for Neurodegenerative Diseases, Berlin, Germany

**Keywords:** Alzheimer’s disease, PLCG2, Phospholipase C gamma 2, Cognitive decline, Mild cognitive impairment

## Abstract

A rare coding variant (rs72824905, p.P522R) conferring protection against Alzheimer’s disease (AD) was identified in the gene encoding the enzyme phospholipase-C-γ2 (*PLCG2*) that is highly expressed in microglia. To explore the protective nature of this variant, we employed latent process linear mixed models to examine the association of p.P522R with longitudinal cognitive decline in 3595 MCI patients, and in 10,097 individuals from population-based studies. Furthermore, association with CSF levels of pTau_181_, total tau, and Aβ_1-42_ was assessed in 1261 MCI patients. We found that MCI patients who carried the p.P522R variant showed a slower rate of cognitive decline compared to non-carriers and that this effect was mediated by lower pTau_181_ levels in CSF. The effect size of the association of p.P522R with the cognitive decline and pTau_181_ was similar to that of *APOE-ε4*, the strongest genetic risk factor for AD. Interestingly, the protective effect of p.P522R was more pronounced in MCI patients with low Aβ_1-42_ levels suggesting a role of PLCG2 in the response to amyloid pathology. In line with this hypothesis, we observed no protective effect of the *PLCG2* variant on the cognitive decline in population-based studies probably due to the lower prevalence of amyloid positivity in these samples compared to MCI patients. Concerning the potential biological underpinnings, we identified a network of co-expressed proteins connecting *PLCG2* to *APOE* and *TREM2* using unsupervised co-regulatory network analysis. The network was highly enriched for the complement cascade and genes differentially expressed in disease-associated microglia. Our data show that p.P522R in *PLCG2* reduces AD disease progression by mitigating tau pathology in the presence of amyloid pathology and, as a consequence, maintains cognitive function. Targeting the enzyme *PLCG2* might provide a new therapeutic approach for treating AD.

**Electronic supplementary material:**

The online version of this article (10.1007/s00401-020-02138-6) contains supplementary material, which is available to authorized users.

## Introduction

Alzheimer’s disease (AD) is highly heritable and the most common cause of neurodegenerative dementias. The discovery of mutations in amyloid precursor protein (*APP*), Presenilin1 and Presenilin2 (*PSEN1/2*) causing rare familial AD laid an important foundation for the ‘*amyloid cascade hypothesis’*, postulating that the aggregation of amyloid is causative for AD and triggers downstream pathological events such as the formation of tau pathology [38]. The importance of amyloid for the development of the more common, sporadic form of AD has recently been emphasized by two large genome-wide association studies (GWAS) [33, 41]. Besides amyloid, GWAS studies suggest additional causative molecular pathways, including immune system processes [33, 35, 41]. Interestingly, it has been suggested that pathways supported by genetic evidence might provide entry points for drug development with a better chance of clinical success [37, 55]. Unfortunately, for most GWAS-identified loci, the variants responsible for the AD-association are unknown, which hampers their translation into a clear functional consequence.

In 2017, we and others identified a specific rare nonsynonymous coding variant (rs72824905, p.P522R) in the gene encoding the enzyme phospholipase-C-γ2 (*PLCG2*) that confers protection against the susceptibility to AD [68]. This association has been replicated in multiple independent samples from different populations [10, 13, 78]. *PLCG2* is an enzyme mainly expressed in immune cells including microglia which are involved in innate immunity [47, 78]. *PLCG2* is related to autoimmune diseases and involved in the activation of platelets in response to amyloid [8, 52, 66]. From a functional perspective, p.P522R induces a slight increase in *PLCG2* activity [47]. Thus, therapeutic strategies similarly modulating *PLCG2* activity as p.P522R may offer a new treatment option for common sporadic AD forms. To achieve this latter aim, we need first to understand the biological mechanisms linking p.P522R in *PLCG2* to the pathophysiological processes of AD. Importantly, van der Lee and colleagues [78] suggested that the p.P522R might also have a protective effect on other neurodegenerative dementias [i.e., Dementia with Lewy Body (DLB) and frontotemporal dementia (FTD)] as well as a positive effect on longevity. However, it is unclear whether these protective effects result from an attenuated protein accumulation process that may be accelerated by microglia activation [4, 5, 28, 70, 72, 79, 85] in carriers of the mutated *PLCG2* gene, or whether the response to accumulated protein aggregates is different, which is the suggested modus operandi for other AD-associated genes like *TREM2* [34].

To understand the boundary conditions and mechanisms of p.P522R effects, we examined its association with cognitive decline in patients with mild cognitive impairment (MCI) and population-based studies. We also analyzed the effect of p.P522R on cerebrospinal fluid (CSF) biomarkers levels amyloid-beta 1-42 (Aβ_1-42_), total tau (tTau) protein, and phosphorylated tau (pTau_181_) protein as well as their interactions in patients with MCI. We also investigated potential biological underpinnings for the effect of PLCG2 within the context of AD pathology. We focused our analysis on the identification of a link between PLCG2 and the known functional relationship between APOE and TREM2. While research identified APOE as a ligand for TREM2 [67, 84] and PLCG2 as an important element of the TREM2 downstream signaling cascade [54], a link between PLCG2 and APOE is currently missing. Consequently, we conducted an unsupervised analysis of trans-co-regulatory network analysis to identify a biological pathway shared between *APOE* and the *TREM2*–*PLCG2-signaling* cascade.

## Methods

### Study cohorts

**Fig. 1:**
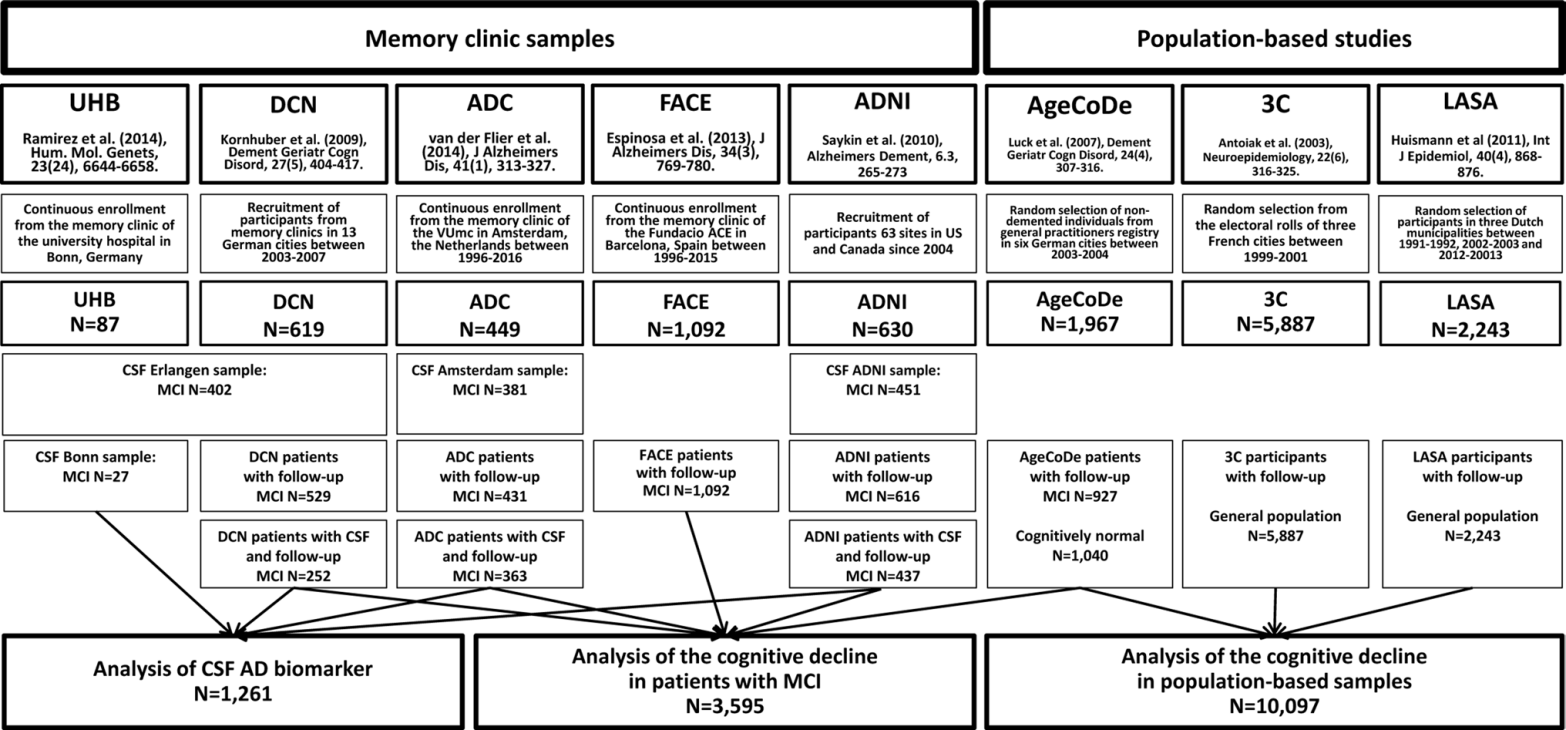
Study design

The design, recruitment, and diagnostic procedures are described in detail in the supplementary text 1, online resource. A flowchart of the participant recruitment and their assignment to outcome-specific analyses is presented in Fig. 1. All participants included in the analyses were older than 50 years, since the focus of our study was the effect of p.P522R in late life.

Longitudinal cognitive decline was analyzed in 3595 MCI patients. These patients were derived from four memory clinic cohorts, namely the Amsterdam dementia cohort (ADC), the German dementia competence network (DCN), the Spanish Fundacio ACE (FACE), and the Alzheimer’s disease neuroimaging initiative (ADNI) cohort that was launched as public–private partnership led by Michael Weiner with the aim to establish measurements of disease progression in MCI and AD (see supplementary text 1, online resource for full description). Also, MCI patients from the prospective, general practitioner-registry-based German study on aging, cognition, and dementia (AgeCoDe) were included. Participants of the AgeCoDe cohort were all non-demented at baseline. Among those, the MCI patients were identified by a standardized diagnostic procedure at each visit and included in this analysis.

10,097 participants from the Three-City Study (3C) and the Longitudinal Aging Study Amsterdam (LASA), as well as all participants from the AgeCoDe study, were included for the analysis of the cognitive decline in population-based samples regardless of MCI or dementia diagnoses.

To analyze the effect of p.P522R on proxy measures of amyloid and tau pathology in the CSF, levels of Aβ_1-42_, pTau_181_, and tTau protein were analyzed. CSF data were collected from 1,261 MCI from the DCN, ADC and ANDI cohort as well as from the memory clinic of the University Hospital of Bonn (UHB) and processed at different centers (supplementary text 4, online resource).

### Genotyping

In all samples, DNA was extracted using standard procedures. In all cohorts except LASA and ADNI, p.P522R was directly genotyped using the MassARRAY (3C cohort, Agena Bioscience), custom content using the Infinium-global-screening-array-24-v1 (ADC cohort, Illumina), or a TaqMan custom design genotyping assay (other cohorts, Thermo Fisher Scientific), respectively. In LASA, p.P522R genotypes were derived from imputation based on the Infinium-global-screening-array-24-v1(Illumina, imputation quality: *R*^2^ = 0.89) or the AXIOM-NL (Affymetrix, imputation quality: *R*^2^ = 0.93) array. In ADNI, three different platforms [i.e., Illumina Human610-Quad BeadChip (imputation quality: *R*^2^ = 0.87), HumanOmniExpress BeadChip (imputation quality: *R*^2^ = 0.83), and Illumina Omni 2.5 M (imputation quality: *R*^2^ = 0.93)] were used for deriving p.P522R genotypes from imputed values. In case multiple platforms were used for genotyping the same individual, we retained p.P522R imputations with the highest genotype probability. All procedures are described in detail in supplementary text 2, online resource. For a fraction of the samples included in the analysis, GWAS data were available. In these samples, all individuals of non-European ancestry were discarded and the presence of population stratification was excluded by visually inspecting a plot of the first two principal components (PCs) of the GWAS data (supplementary Fig. 1a–g, online resource). No evidence for population stratification was found during visual inspection. In line with that, adjustment for PCs in those samples with GWAS data did not change the effects observed for p.P522R (supplementary text 3). For this reason, PCs were not used to adjust the analysis maximizing thereby the data set for analysis and thus increasing the statistical power.

### CSF collection and biomarker measurement

CSF levels of Aβ_1-42_, pTau_181_, and tTau were measured using commercial ELISA immunoassays. Samples were quantified in different laboratories (CSF samples from Erlangen, Bonn, Amsterdam, and the ADNI cohort, see Fig. 1) using different methods described in supplementary text 4.1, online resource. For the present analysis, CSF from different cohorts was harmonized using the method described by Zhou and colleagues [89] (supplementary text 4.2, online resource).

### Neuropsychological assessments

In all cohorts, the Mini-Mental State Examination (MMSE) was used as this measure of global cognition is sensitive to change in patients with MCI [59] and available in all cohorts. In the population-based studies, the MMSE might show ceiling effects. Therefore, additional measurements were used for the cognitive function: the 3C cohort also measured episodic memory using the Benton visual retention test, the LASA cohort used the Dutch version of the Adult Verbal Learning Task, and the AgeCoDe cohort used the CERAD word list learning task with delayed recall. Besides, verbal fluency was assessed in the 3C and AgeCoDe study using the Isaac set test and the CERAD animal fluency task, respectively. References and further descriptions for all tests are given in supplementary text 5, online resource.

### Statistical analysis

Statistical analyses were conducted in R version 3.4.4 [58] and Mplus version 7.31 [50]. References to all utilized software are provided in supplementary Table 1, online resource. More detailed descriptions of the used methods are provided below and in supplementary text 6, online resource. All *p* values < 0.05 (two-sided) were considered significant.

### Analysis of cognitive decline

The analysis of the effect of p.P522R on longitudinal cognitive decline was performed using latent process linear mixed models [56] (supplementary text 6.1, online resource) as implemented in the R package LCMM [57]. These models jointly estimate a latent process representing the true change of cognition over time and a link function that relates this process to the observed cognitive measurements. This link function takes into account the unequal interval scaling that commonly occurs for cognitive tests and may introduce bias [56] by modeling a monotone but possibly non-linear relationship between the latent process and the outcome. Modeling of the link function also accounted for the skewed distribution of the MMSE indicated ceiling effects in some MCI patients. Moreover, residuals derived from the standard linear mixed model showed deviations from normality which were adjusted by including the appropriate link function. Potential non-linear decline trajectories were assessed using polynomial order of time. The most appropriate link function and polynomial of time were chosen according to the lowest Bayesian information criterion (BIC). Statistical significance of the effect of p.P522R on the decline was assessed using multivariate Wald tests.

For the analysis of MCI patients, median-centered time from MCI diagnosis was used as the time scale to approximate disease progression after cognitive symptom onset. In the AgeCoDe cohort, the baseline for our analysis was defined as the first visit where MCI criteria were met and only subsequent observations were considered as follow-up assessments. In a sensitivity analysis, the follow-up time was restricted up to 6 years to rule out that a possible effect of p.P522R was artificially induced by only a few observations with long follow-up. An integrative data analysis was performed by pooling data from all memory clinic cohorts to maximize the number of p.P522R carriers as recommended for rare predictors [11]. Also, stratified analyses in each cohort were performed. The relationship between p.P522R and *APOE-ε4* was investigated by examining the interaction effect on the cognitive decline between the two genotypes. In addition, effect sizes on cognitive change of p.P522R in the absence and presence of *APOE-ε4* were compared with the effect of *APOE-ε4* alone. Due to potential non-linear decline, effects were calculated at multiple time points on the scale of the latent process, standardized by the predicted variation at the last time point (supplementary text 6.2, online resource).

For the analysis of the effect of p.P522R in three population-based cohorts, median-centered age at assessment was used as the time scale of the linear mixed models representing general age-related cognitive change. Herein, analyses were performed separately for each cohort.

All analyses in all samples were adjusted for age at baseline, as well as gender, education (i.e., dichotomous variable indicating participation in secondary education), and *APOE-ε4* status (supplementary text 6.5, online resource). In the pooled analysis of MCI patients from memory clinic cohorts, analyses were additionally adjusted for cohort. Furthermore, center was included as an additional covariate in the 3C study and the genotyping platform was used as a covariate in LASA. In the case of significant associations, analyses were repeated without adjustment to examine the sensitivity of the results to covariate selection. Missing data in the cognitive outcomes were handled using maximum-likelihood estimation [15], so that no participants had to be excluded due to drop-out.

### Analysis of CSF biomarkers

For CSF analysis, continuous harmonized CSF biomarker data were log-transformed. Robust regression analysis was used to minimize the influence of outlying observations in the data by applying a down-weighting algorithm [40].

AD biomarkers were analyzed in a joint regression model using data from all CSF samples to maximize the number of available p.P522R carriers. Also, stratified analyses were performed in the samples from the three European memory clinic cohorts (DCN, ADC, and UHB) and the ADNI cohort. All regression analyses were controlled for age, gender, the origin of the CSF samples, and the presence of at least one *APOE-ε4* allele (see supplementary text 6.5, online resource). We adjusted *p* values from tests of multiple outcomes (i.e., three different AD biomarkers) by applying the Bonferroni–Holm correction.

To explore whether p.P522R affects the interplay of AD biomarkers, four AD biomarker categories were constructed as described by Jack and colleagues [29] based on laboratory-specific cut-offs. These categories were derived: AD (Abeta_1-42_ positive and pTau_181_ positive, irrespective of tTau), AD pathologic change (Abeta_1-42_ positive and pTau_181_ negative, irrespective of tTau), non-AD pathologic change (Abeta_1-42_ negative and pTau_181_ positive and/or tTau positive), and no pathologic change (all three biomarkers negative). These categories were used as the outcome in a multinomial regression model with AD as the reference category and Bonferroni–Holm correction for multiple testing (i.e., comparison of four biomarker categories).

In addition, a varying coefficient generalized additive model [82] (supplementary text 6.3, online resource) was used to test whether the effect of p.P522R on continuous pTau_181_ and tTau levels differs across different Aβ_1-42_ levels. Thin plate regression splines [81] were used to allow for non-linear, potentially sigmoid relationships between AD biomarkers as proposed by Jack and colleagues [30]. Posterior simulation with simultaneous confidence intervals was used to identify those levels of CSF Aβ_1-42_ at which p.P522R show its strongest effects.

All analyses in CSF samples were adjusted for age, gender, CSF samples, and *APOE*-ε4 status (supplementary text 6.5, online resource). In a sensitivity analysis, all analyses were repeated without adjustment for covariates.

### Mediation analysis using structural equation models

To evaluate whether or not the associations of p.P522R with CSF biomarkers underlie the association of p.P522R with cognitive decline, a mediation analysis was conducted using structural equation modeling in Mplus version 7.31 [50]. Herein, 1052 MCI patients from the DCN, ADC, and ADNI cohorts were included only if they had CSF AD biomarkers as well as longitudinal MMSE assessments available. To assess mediation, an indirect effect of p.P522R on the cognitive change in the MMSE over 4 years via Aβ_1-42_ and tTau were estimated, as well as interaction effects between p.P522R, and Aβ_1-42_ or pTau_181_. Analyses were repeated using pTau_181_ instead of tTau in the models. Additional details are provided in supplementary text 6.4, online resource.

### Co-regulatory network analysis

The GeneFriends tool was used to generate an unsupervised co-expression gene map based on 4164 Human Microarray data sets containing 26,113 experimental conditions and 19,080 genes. We decided to use the microarrays data available in GeneFriends tool, because the number of experimental conditions available was larger and better annotated compared to the RNA-sequencing data available in the same tool. Further description of the microarray methods is provided elsewhere [77]. Of note, loci around the target genes might co-express due to the existence of common regional co-regulation motifs. Cis-co-regulated genes were deleted from the respective list by removing all transcripts located 200 kb around *PLCG2*, *APOE*, and *TREM2* loci. Hence, only highly trans-co-regulated and positively co-expressed genes that are collectively upregulated were selected for further analysis (co-expression value > 0.5). Next, the WebGestalt tool [45] was used to identify potential enrichments in identified co-regulated gene lists using overrepresentation enrichment analyses of Gene Ontology non-redundant biological process in humans. Furthermore, genes co-expressed with *PLCG2* were tested for enrichment of shared co-expression with *APOE* and *TREM2* using Fisher’s exact test (supplementary text 6.6, online resource). Genes co-expressed in all three candidate genes were selected for additional enrichment analyses using STRING [75] and WebGestalt. Finally, this shared gene set was tested for enrichment of genes differentially expressed in cell-type-specific biomaterials derived from brain, i.e., microglia of AD patients [49] as well as microglia derived from the 5XFAD AD mouse model [39] and the hMAPT-P301S model for tauopathies [19] (supplementary text 6.6, online resource).

## Results

### The p.P522R variant is associated with slower cognitive decline in MCI patients

**Fig. 2:**
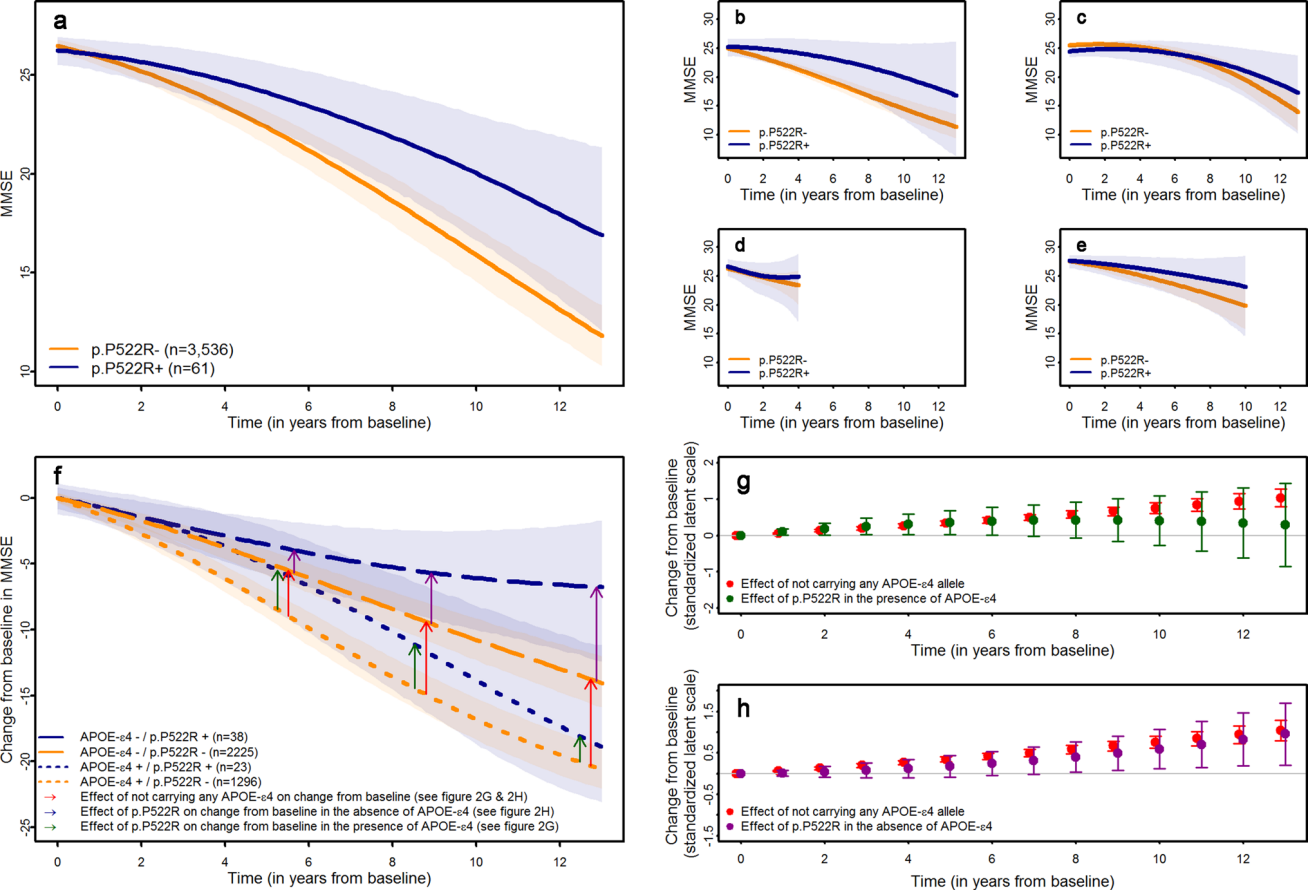
Effect of p.P522R on the cognitive decline in 3,595 MCI patients. **a** Predicted trajectories of cognitive decline for p.P522R carrier and non-carrier in the pooled sample of all MCI patients. **b** Predicted trajectories of cognitive decline for p.P522R carrier and non-carrier in the Fundacio ACE (FACE) cohort. **c** Predicted trajectories of cognitive decline for p.P522R carrier and non-carrier in the German study on aging, cognition, and dementia (AgeCoDe) cohort. **d** Predicted trajectories of cognitive decline for p.P522R carrier and non-carrier in the Dementia competence network (DCN) cohort. **e** Predicted trajectories of cognitive decline for p.P522R carrier and non-carrier in the ADNI cohort. Predicted trajectories for the Amsterdam dementia cohort (ADC) are displayed in supplementary figure 1, online resource due to the unreasonably low number of p.P522R carriers with sufficient follow-up. **f** Effect of p.P522R and APOE-ε4 on the cognitive change from baseline in the MMSE derived from a linear mixed model with a latent process including an interaction term between APOE-ε4 and p.P522R. Differences on the latent process scale between APOE-ε4 carrier and non-carrier who do not carry the pP522R variant were derived to assess the effect size of APOE-ε4 (red arrow in Fig. 2f and red dots in Fig. 2g, h). The difference between p.P522R carrier and non-carrier in the absence (magenta arrows in Fig. 2f and magenta dots in Fig. 2g) and the presence of APOE-ε4 (green arrows in fig. 2f and green dots in Fig. 2h) were calculated, as well. **g** The effect size of the association of not carrying any APOE-ε4 allele (red dots) and p.P522R in the presence of APOE-ε4 (green dots) with the cognitive change at different time points. Effect sizes are displayed on the scale of the latent process of the mixed model standardized by the expected variance of the latent process at the last time point considered (see supplementary text 6.2, online resource). **h** The effect size of the association of not carrying any APOE-ε4 allele (red dots) and p.P522R in the absence of APOE-ε4 (magenta dots) with the cognitive change at different time points. Effect sizes are displayed on the scale of the latent process of the mixed model standardized by the expected variance of the latent process at the last time point considered (see supplementary text 6.2, online resource)

Due to the low frequency of the rare variant, it is unlikely to detect statistically significant effects in the individual cohorts. We, therefore, pooled data from all cohorts (Table 1) in a joint analysis in a first step. Here, carriers of the p.P522R variant (*n* = 61) showed a slower cognitive decline than non-carriers (*χ*^2^(2) = 7.83, *p* = 0.020, Fig. 2a; supplementary Table 2, online resource). Importantly, analyses stratified for cohort demonstrated a highly consistent protective effect of p.P522R in each of the samples (Fig. 2b–e, supplementary Fig. 2, and supplementary Table 3, online resource). This consistency was further confirmed by a non-significant interaction between p.P522R and cohorts concerning the effect on the cognitive decline (*χ*^2^(8) = 3.55, *p* = 0.895). Sensitivity analyses showed no change in the association without adjustment for covariates (*χ*^2^(2) = 8.95, *p* = 0.011) or using a restricted follow-up interval of 6 years (*χ*^2^ (2) = 6.58, *p* = 0.037, supplementary Table 2, online resource).

**Table 1:** Characteristics of MCI samples included in the analysis of the cognitive decline

	FACE		AgeCoDe		DCN		ADC		ADNI		Total	
	p.P522R carrier	p.P522R non-carrier	p.P522R carrier	p.P522R non-carrier	p.P522R carrier	p.P522R non-carrier	p.P522R carrier	p.P522R non-carrier	p.P522R carrier	p.P522R non-carrier	p.P522R carrier	p.P522R non-carrier
Sample size (*n *(% of total sample))	12 (1.1%)	1080 (98.9%)	26 (2.8%)	901 (97.2%)	9 (1.7%)	520 (98.3%)	4 (0.09%)	427 (99.1%)	10 (1.6%)	606 (98.4%)	61 (1.7%)	3534 (98.3%)
Age (m (SD))	77.56 (5.02)	76.64 (6.91)	81.15 (4.58)	82.31 (4.72)	65.78 (8.63)	66.68 (8.35)	65.63 (4.47)	66.99 (7.40)	72.61 (7.55)	73.32 (7.50)	75.76 (8.34)	74.89 (8.92)
Female gender (*n *(%))	10 (83.3%)	692 (64.1%)	18 (69.2%)	587 (65.1%)	3 (33.3%)	224 (43.1%)	1 (25.0%)	144 (33.7%)	6 (60.0%)	233 (38.4%)	38 (62.3%)	1880 (53.2%)
High education (*n *(%))	2 (16.7%)	245 (22.7%)	18 (69.2%)	509 (56.5%)	5 (55.6%)	231 (44.4%)	2 (50.0%)	293 (68.6%)	8 (80.0%)	520 (85.8%)	35 (57.4%)	1798 (50.9%)
APOE-ε4 carrier (*n *(%))	4 (33.3%)	364 (33.7%)	8 (30.8%)	223 (24.8%)	6 (66.7%)	191 (36.7%)	2 (50.0%)	223 (52.2%)	3 (30.0%)	298 (49.1%)	23 (37.7%)	1299 (36.8%)
MMSE at baseline (m (SD))	25.58 (2.81)	25.49 (2.90)	25.35 (2.43)	26.19 (2.12)	27.56 (1.88)	27.21 (2.07)	25.50 (3.11)	26.74 (2.18)	27.60 (1.51)	27.66 (1.76)	26.10 (2.49)	26.45 (2.47)
Mean observation time (m (SD), in years)	5.07 (3.39)	5.06 (3.08)	5.83 (4.27)	4.16 (3.53)	2.51 (0.83)	1.83 (1.12)	0.84 (1.44)	2.44 (2.04)	5.73 (2.07)	4.12 (2.46)	4.85 (3.60)	3.88 (3.02)

High education was operationalized as participation in secondary education

*MMSE* mini-mental state examination, *n(%)* number of individuals and percent within group, *m *mean, *SD* standard deviation

In the same cohorts, the APOE-ε4 allele was, as expected, associated with accelerated cognitive decline (*χ*^2^ (2) = 138.33, *p* = 9.27 × 10^–37^, supplementary Table 2, online resource), but there was no statistically significant interaction between APOE-ε4 and p.P522R (*χ*^2^ (2) = 2.87, *p* = 0.238, supplementary table 4, online resource). The absence of a significant interaction may arise from limited statistical power due to the small number of carriers for both the p.P522R and the APOE-ε4 alleles (*n* = 23). However, the protective effect of p.P522R and the detrimental effect of APOE-ε4 on the cognitive change from baseline were similar in magnitude though in opposite directions (Fig. 2f). To directly compare the protective effect of *PLCG2* with that of *APOE*, we expressed the *APOE* effect on cognitive decline in protective terms, i.e., cognitive changes when APOE-ε4 is absent (Fig. 2g, h). This analysis suggested that the protective effect of p.P522R is as strong as the detrimental APOE-ε4 effect during the first 6 years of follow-up, but this effect decreased at later follow-ups (Fig. 2f, g). This raises the possibility that p.P522R may initially counteract, at least in part, the detrimental effect of the APOE-ε4 allele on cognitive function and could delay deterioration in MCI patients for several years. We also found that the protective effect on cognitive change associated with p.P522R in APOE-ε4 non-carriers was similar to the reduced cognitive change observed in the absence of APOE-ε4 alone, especially at later stages of the cognitive trajectory (Fig. 2h).

### Age-related cognitive decline in population-based samples is not associated with p.P522R

Given our observation in MCI and the previously reported effect on longevity [78], we sought to explore whether the effect of p.P522R on cognitive decline can be also extended to the general population (Table 2). As expected from previous case–control studies [68, 78], the frequency of p.P522R in the population-based samples was significantly higher than in the sample of MCI patients from memory clinic cohorts (i.e., FACE, DCN, ADNI, and ADC) who are an at-risk population for dementia (*χ*^2^(1) = 10.02, *p* = 0.002). Importantly, the statistical difference found for this variant between the MCI samples and the population-based studies further support the protective effect of *PLCG2*. However, p.P522R did not modulate the cognitive decline in any of the neuropsychological tests explored in the population-based samples (supplementary table 5, supplementary Figs. 3–5, online resource).

**Table 2:** Characteristics of population-based samples included in the analysis of the cognitive decline

	AgeCoDe		3 City study		LASA	
	p.P522R carrier	p.P522R non-carrier	p.P522R carrier	p.P522R non-carrier	p.P522R carrier	p.P522R non-carrier
Sample size (*n *(% of total sample))	49 (2.5%)	1918 (97.5%)	127 (2.2%)	5760 (97.8%)	56 (2.5%)	2187 (97.5%)
Age (m (SD))	79.71 (3.64)	79.56 (3.51)	73.98 (5.22)	74.22 (5.46)	63.47 (6.39)	64.79 (7.63)
Female gender (*n *(%))	32 (65.3%)	1245 (64.9%)	81 (63.8%)	349 (60.6%)	34 (60.7%)	1159 (53.0%)
High education (*n *(%))	23 (46.9%)	848 (44.2%)	49 (38.6%)	3030 (52.6%)	45 (80.4%)	1619 (74.0%)
APOE-ε4 carrier (*n *(%))	10 (25.6%)	410 (21.4%)	33 (26.0%)	1188 (20.7%)	18 (29.6%)	638 (28.8%)
MMSE at baseline (m (SD))	26.88 (2.34)	27.41 (1.95)	27.34 (1.96)	27.37 (1.92)	27.64 (2.36)	27.88 (2.01)
Mean observation time (m (SD), in years)	6.05 (4.08)	6.21 (4.03)	5.40 (2.28)	5.34 (2.36)	11.72 (7.62)	10.73 (7.05)

High education was operationalized as participation in secondary education

*MMSE* mini-mental state examination, *n(%)* number of individuals and percent within group, *m* mean, *SD* standard deviation

### p.P522R is associated with reduced levels of CSF pTau_181_ and tTau

**Fig. 3:**
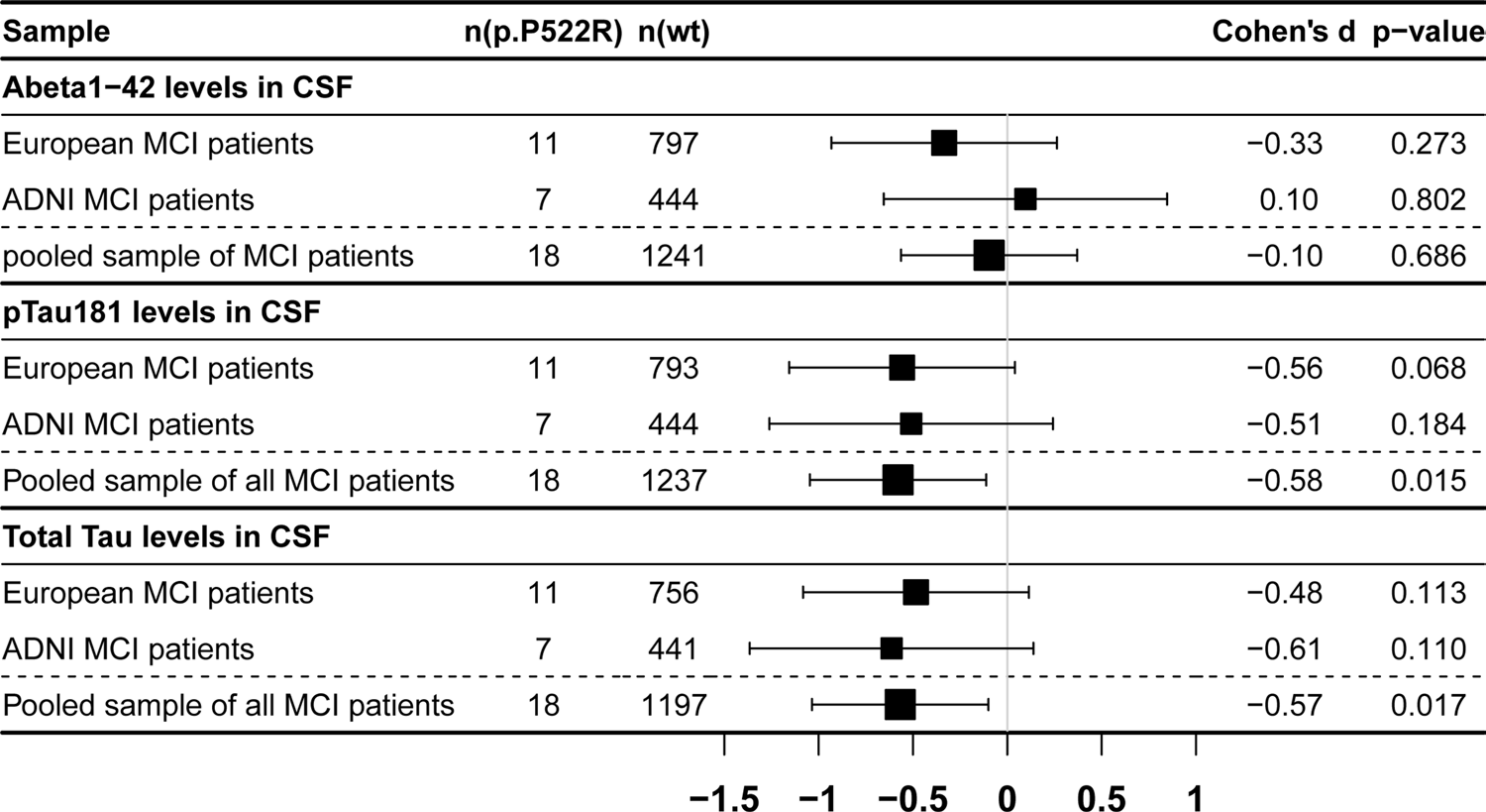
Associations of p.P522R with CSF biomarkers of Alzheimer’s disease (AD). Black dots and arrows represent Cohen’s d estimates and 95% confidence interval, respectively. For Aβ_1-42_, negative Cohen’s d estimates indicate more pathology. In the case of pTau_181_ and tTau, positive Cohen’s d values represent more pathology. *N(p.P522R)* number of p.P522R carrier, *n(wt)* number of p.P522R non-carrier, *Aβ*_*1-42*_ amyloid-beta 1-42, *pTau*_*181*_ phosphorylated tau

To examine the etiology underlying the protective effect of p.P522R, we analyzed AD-related CSF biomarkers in MCI patients (Table 3). Carriers of the p.P522R variant (*n* = 18) showed a reduction of pTau_181_ (Est(SE) = −  0.12(0.05), *p* = 0.015, *d* = − 0.58) and tTau levels (Est(SE) = − 0.12 (0.05), *p* = 0.017, *d* = − 0.57) in the pooled sample of all MCI patients. The association remained statistically significant after Bonferroni–Holm correction for multiple testing (Fig. 3, supplementary table 6, online resource). In contrast, there was no association of p.P522R with Aβ_1-42_ levels (Est(SE) = − 0.02(0.04), *p* = 0.686, *d* = − 0.10). The effect of p.P522R was consistent across MCI patients from both the European MCI cohorts (DCN, ADC, and UHB) and the ADNI cohort (Fig. 3). This was confirmed by non-significant interactions between p.P522R and CSF samples (supplementary table 7, online resource). Sensitivity analyses suggested that unadjusted analysis led to a similar pattern of results (supplementary table 6, online resource).

**Table 3:** Characteristics of samples included in the analysis of CSF AD-biomarkers

	UHB		DCN		ADC		ADNI		Total	
	p.P522R carrier	p.P522R non-carrier	p.P522R carrier	p.P522R non-carrier	p.P522R carrier	p.P522R non-carrier	p.P522R carrier	p.P522R non-carrier	p.P522R carrier	p.P522R non-carrier
Sample size (*n *(% of total sample))	1 (1.1%)	86 (98.9%)	8 (2.3%)	334 (97.7%)	2 (0.5%)	379 (99.5%)	7 (1.6%)	444 (98.4%)	18 (1.4%)	1243 (98.6%)
Age (m (SD))	63.00 (0.00)	66.83 (8.58)	67.88 (10.11)	66.55 (8.17)	64.65 (3.18)	67.03 (7.72)	74.16 (5.36)	72.75 (7.54)	69.69 (8.26)	68.93 (8.34)
Male gender (*n *(%))	1 (100%)	53 (61.6%)	6 (75.0%)	191 (57.2%)	2 (100%)	251 (66.2%)	2 (28.6%)	272 (61.3%)	11 (61.7%)	767 (61.7%)
APOE-ε4 carrier (*n *(%))	0 (0.0%)	41 (47.7%)	5 (62.5%)	138 (41.3%)	2 (100%)	194 (51.2%)	3 (42.9%)	211 (47.7%)	10 (55.6%)	584 (47.0%)
CSF Aβ_1-42_ levels (m (SD) in pg/mL)^a^	354.86 (0.00)	736.09 (337.80)	615.75 (308.64)	759.47 (344.36)	719.00 (172.08)	732.18 (345.58)	711.34 (204.54)	717.03 (326.14)	649.90 (337.89)	734.37 (252.03)
CSF pTau_181_ levels (m (SD) in pg/mL)^b^	52.51 (0.00)	67.42 (34.19)	40.58 (9.44)	65.58 (34.70)	72.46 (28.12)	66.02 (34.75)	56.52 (24.09)	71.84 (37.70)	50.98 (20.15)	68.08 (35.86)
CSF total tau levels (m (SD) in pg/mL)^c^	227.21 (0.00)	464.46 (335.86)	269.88 (80.76)	452.35 (415.61)	548.35 (281.39)	420.87 (225.87)	326.83 (164.79)	436.35 (257.94)	320.60 (157.39)	437.03 (304.20)
Pathological Aβ_1-42_ CSF levels (*n *(%))^a^	1 (100%)	26 (30.2%)	5 (62.5%)	132 (39.5%)	1 (50.0%)	194 (51.2%)	5 (71.4%)	290 (65.3%)	12 (66.7%)	642 (51.6%)
Pathological pTau_181_ CSF levels (*n *(%))^b^	0 (0.0%)	42 (48.8%)	0 (0.0%)	148 (44.3%)	2 (100%)	232 (61.2%)	5 (71.4%)	325 (73.1%)	7 (38.9%)	747 (60.1%)
Pathological total tau CSF levels (*n *(%))^c^	0 (0.0%)	27 (31.4%)	3 (37.5%)	198 (59.3%)	2 (100%)	197 (52.0%)	2 (28.6%)	161 (36.3%)	7 (38.9%)	583 (46.9%)

*Aβ*_*1-42*_ amyloid-beta 1-42, *pTau*_*181*_ phosphorylated tau, *CSF* cerebrospinal fluid, *n(%)* number of individuals and percent within group, *m* mean, *SD* standard deviation

^a^Sample size *n* = 1259

^b^Sample size *n* = 1255

^c^Sample size *n* = 1215

Again, the effect sizes of p.P522R and APOE-ε4 on pTau_181_ in the pooled MCI patients were similar, but in opposite directions (p.P522R: *d* = − 0.58, APOE-ε4: *d* = 0.56; supplementary table 8, online resource). We did not observe a statistically significant interaction between APOE-ε4 and p.P522R regarding any CSF biomarker (supplementary table 9, online resource). The lack of interaction may, again, follow the same power issue derived from the small-sample size of carriers of both variants (*n* = 10). Notably, APOE-ε4 showed an effect on all CSF biomarkers, while p.P522R only had an effect on pTau_181_ and tTau levels (supplementary table 8, online resource).

### p.P522R exerts its strongest effect downstream of amyloid pathology

To further explore the role of p.P522R in AD, we examined the interplay of the same CSF biomarkers using the AD biomarker categories proposed by Jack and colleagues [29]. A multinomial regression model using the AD category (Aβ_1-42_ and pTau_181_ positive, *n* = 522) as a reference revealed that p.P522R was associated with the presence of AD pathologic change (Aβ_1-42_ positive, pTau_181_ negative, OR(95% CI) 6.28(3.3, 11.9), *p* = 0.004, *n* = 279) but not with the presence of non-AD pathologic changes (Aβ_1-42_ negative, pTau_181_ and/or tTau positive, OR(95% CI) 0.93(0.39, 2.24), *p* = 0.938, *n* = 129) or normal CSF biomarkers (all three biomarkers negative, OR(95% CI) 1.81(0.86, 3.83), *p* = 0.425, *n* = 323). Noteworthy, the small number of amyloid-negative individuals might have limited the statistical power to detect an association of p.P522R with the presence of non-AD pathological changes. Unadjusted analyses revealed the same results (supplementary table 10, online resource).

**Fig. 4:**
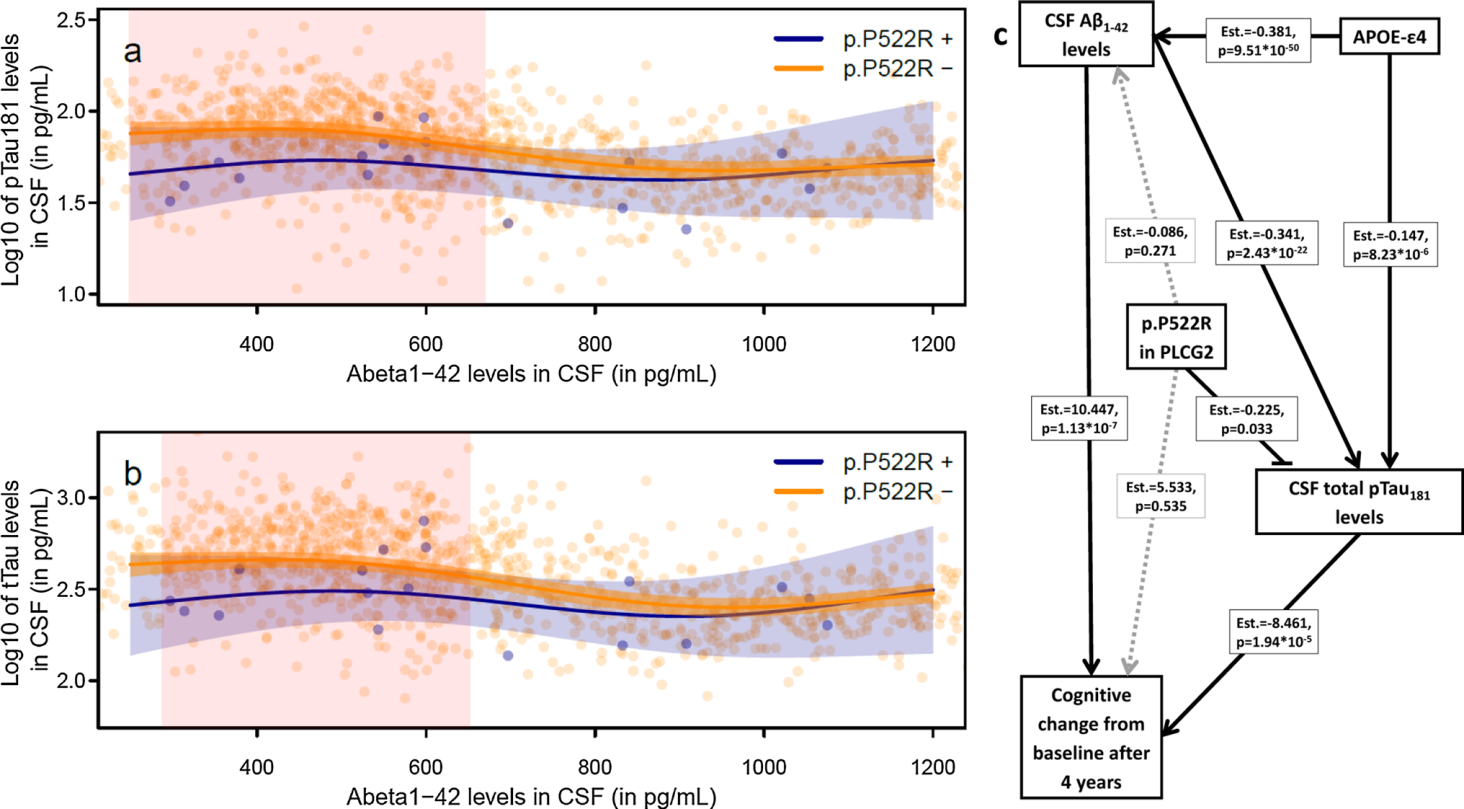
Role of p.P522R in the interrelationship between amyloid pathology, neurodegeneration, and cognitive decline. **a** Predicted relationship between Aβ_1-42_ levels in CSF and pTau_181_ levels in CSF for p.P522R carrier and non-carrier. Red shaded areas indicate significant differences between p.P522R carrier and non-carrier. Significance is based on the estimation of simultaneous confidence intervals that consider the statistical testing at multiple CSF levels. **b** Predicted relationship between Aβ_1-42_ levels in CSF total tau levels in CSF for p.P522R carrier and non-carrier. Red shaded areas indicate significant differences between p.P522R carrier and non-carrier. Significance is based on the estimation of simultaneous confidence intervals that consider the statistical testing at multiple CSF levels. **c** Results from a structural equation model assessing whether the effect of p.P522R on the cognitive change in the normalized MMSE (range 0–100) is mediated Abeta_1-42_ levels or pTau_181_ levels in CSF. The model showed a good fit to the data (Model fit indices: RMSEA = 0.017, CFI = 0.996, SRMR = 0.060, see supplementary text 6.4, online resource)

We also applied generalized additive models in the MCI data set to model the influence of the non-linear relationship of Aβ_1-42_ with pTau_181_ and tTau in the CSF on the effect of p.P522R. We observed that the effect of p.P522R on pTau_181_ and tTau levels is significantly stronger when CSF levels of Aβ_1-42_ are low (i.e., more abnormal, pTau_181_: *p* = 0.013, tTau: *p* = 0.020, Fig. 4a, b, supplementary table 11, online resource).

We then sought to conduct a mediation analysis in 1052 MCI patients with both CSF biomarkers and longitudinal follow-up data using structural equation modeling (Fig. 4c, supplementary table 12, online resource). This analysis will allow establishing a link between the findings derived from the cognitive decline in MCI and those obtained from the CSF. This strategy revealed that the effect of p.P522R on the change in cognition from baseline over 4 years was mediated by pTau_181_ (Est = 1.91, 95% CI 0.12, 4.11) but not by Aβ_1-42_ (Est = 0.84, 95% CI − 0.54, 2.63, supplementary table 13, online resource). Noteworthy, we found a significant interaction of p.P522R and Aβ_1-42_ levels on the cognitive decline (*χ*^2^(2) = 8.86, *p* = 0.012), indicating that the protective p.P522R variant is associated with a less pronounced cognitive decline at more abnormal levels of Aβ_1-42_ (supplementary table 14, supplementary Fig. 6, online resource). This observation is in line with the findings obtained from the generalized additive model. There was no interaction effect between p.P522R and pTau_181_ levels (*χ*^2^(2) = 0.68, *p* = 0.712) on the cognitive decline. The pattern of results was similar when tTau instead of pTau_181_was used as a mediator. However, the effect of p.P522R on tTau (Est(SE) = − 0.22(0.12), *p* = 0.051) and the corresponding mediation effect on the cognitive change over 4 years (Est = 1.94, 95% CI = − 0.13, 4.07) showed only a trend-level association (supplementary tables 12–14, online resource).

Conversely, we observed that the effect of APOE-ε4 on cognition change from baseline over 4 years is mediated by both, Aβ_1-42_ (Est = − 4.14, 95% CI − 5.67, − 2.57) and pTau_181_ (Est = − 1.24, 95% CI − 2.15, − 0.56). However, there were no interaction effects between APOE-ε4 and the CSF levels of Aβ_1-42_ (*χ*^2^(2) = 1.29, *p* = 0.526) and pTau_181_ (*χ*^2^(2) = 2.531, *p* = 0.282) regarding the cognitive decline.

### *APOE* shares a common co-regulatory network with *PLCG2* and *TREM2*

**Fig. 5:**
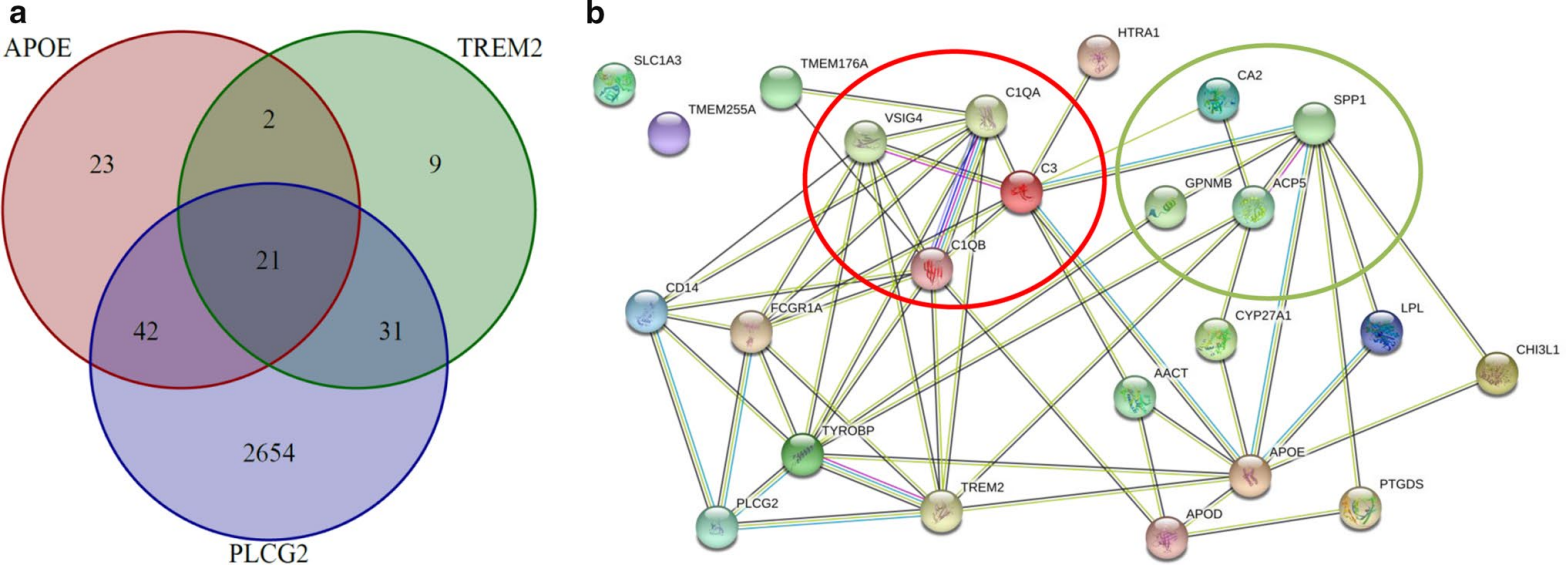
Co-regulatory network shared between APOE, TREM2, and PLCG2. **a** Venn diagram showing the number and the overlap of genes highly co-expressed APOE, TREM2, and PLCG2. **b** Depiction of potential relationships between genes included in the shared co-expression network of APOE, TREM2, and PLCG2. The red circle marks members of the complement cascade. The green circle marks genes involved in tissue remodeling. Green lines between proteins indicate evidence for interaction based on text mining, black lines represent co-expression, blue lines indicate evidence from curated databases, and magenta lines indicate experimentally conformed interactions

To explore the biological underpinnings of the interplay of *APOE* with *PLCG2* and the membrane receptor *TREM2*, we searched for co-regulated genes and pathways. An unsupervised search identified 2748 genes co-expressed with *PLCG2* showing enrichment of immune-related pathways (supplementary table 15, online resource). In our analysis, gene-specific networks of *APOE* and *TREM2* were also highly enriched for immunological function (supplementary tables 16–17, online resource). Genes co-expressed with *PLCG2* were significantly enriched among those co-expressed with *APOE* (*p* = 7.49 × 10^–34^) or *TREM2* (*p* = 1.37 × 10^–33^). Furthermore, *PLCG2*-related genes were disproportionally more likely to be co-expressed with both *APOE* and *TREM2* (*p* = 3.76 × 10^–16^). Noteworthy, the shared gene set of 21 loci simultaneously co-regulated with *APOE*, *TREM2,* and *PLCG2* (Fig. 5) is highly enriched for biological processes related to immune system processes including the complement cascade activation but also tissue remodeling (supplementary table 18, online resource). Furthermore, we also detected a correspondence between the identified network and microglial gene expression in the brain. Herein, we found enrichment for genes differential expressed in microglia from human AD patients (*p* = 3.57 × 10^–12^, [49]) as well as in microglia from mouse models of AD (*p* = 9.47 × 10^–8^, 5XFAD model [39]) or tauopathies (*p* = 2.18 × 10^–6^, hMAPT-P301S model [19], supplementary table 19, online resource).

## Discussion

In this study, we provide the first evidence linking the protective effect of the p.P522R variant in *PLCG2* with a slower cognitive decline in patients with MCI and reduced pTau_181_ and tTau levels. Interestingly, this effect seems to be downstream of Aβ_1-42_ pathology. Furthermore, we showed that *PLCG2* shares a biological co-regulation network with the *APOE* and *TREM2* that is enriched for complement cascade processes and markers of disease-associated microglia. Taken together, our findings strongly support a role of p.P522R in the physiological response to abnormally folded proteins, such as amyloid, and help to characterize the specific function of PLCG2 within the amyloid cascade.

To date, except for *CLU* and *APOE*, few of the identified genetic risk variants in case–control GWAS for AD have shown a consistent effect on disease progression in MCI patients [42]. Our study now adds to this list *PLCG2* as a strong protector of cognitive function at the MCI stage. This effect is mediated by reduced pTau_181_ pathology but not by Aβ_1-42_ pathology. Thus, the p.P522R variant ameliorates cognitive decline by acting downstream of amyloid accumulation, making this accumulation less detrimental. This hypothesis receives additional supports from the observation that p.P522R displays its strongest effect on tau pathology and cognitive decline when amyloid pathology is present. Recent research has further shown that tau pathology depends on Aβ_1-42_-evoked neuroinflammation [28]. Likewise, the neuronal protection conferred by p.P522R in AD may also operate in neurodegenerative diseases caused by the accumulation of other protein aggregates which trigger downstream damaging effects via neuroinflammation. Supporting this hypothesis, p.P522R in *PLCG2* might also have a protective effect on DLB and FTD [78]. For all three diseases (i.e., AD, FTD, and DLB), a genetic overlap has been reported suggesting shared pathogenic pathways which may include pathways related to *PLCG2* [16, 24, 36, 64]. Furthermore, patients with DLB frequently show amyloid pathology [53] that contributes to fast disease progression and cognitive impairment [1] suggesting that microglial reaction to amyloid pathology could mediate the protective role of p.P522R on both AD and DLB. In contrast, amyloid pathology is rarely observed in FTD indicating a slightly different mechanism. However, mutations in *TREM2* have been consistently reported in FTD patients involving, thus, the TREM2 signal cascade in the pathological events occurring in FTD [20, 61]. Besides, functional studies in genes involved in FTD have shown that several of these genes can modulate microglia function either by increasing production of neurotoxic factors and neuroinflammation, or by altering microglial phagocytosis and related degradation pathways [22]. For example, research has shown that levels of soluble TREM2 (sTREM2) in CSF are increased in familial FTD cases carrying mutations in the progranulin gene (*GRN*) compared to controls [83]. Likewise, a C9orf72-deficient mouse showed increased expression of *Trem2*, *C1qa*, and *Tyrobp* linking decreased expression of C9orf72 with microglia activity and age-related neuroinflammation [43]. As with *GRN* and *C9orf72*, other FTD genes, including TANK-binding kinase 1 (*TBK1*), Optineurin (*OPTN*), sequestosome (*SQSTM1*), and Valosin Containing Protein (*VCP*), have been linked to neuroinflammation and microglial function, because these genes can regulate one of the crucial regulators of glial activation and neuroinflammation, the nuclear factor-kappa beta (NF-κβ) [22]. Importantly, NF-κβ has been shown to be a downstream effector of PLCG2 activation [65]. It is, therefore, tempting to speculate that downstream neuroinflammatory processes activated by protein aggregation in FTD will lead to activation of a signaling cascade involving PLCG2 wherein the p.P522R-carrying PLCG2 may show its protective effect. In addition, all these findings together may have uncovered a more general effect of PLCG2 in the response to misfolded protein aggregation found in neurodegenerative disorders. At this point, it is important to note that the reported effect of p.P522R on susceptibility to FTD and DLB still requires validation in independent samples [78].

Besides the effect downstream of Aβ_1-42_ pathology, p.P522R might also contribute to the initial formation and amplification of pathological protein aggregates itself instead of modulating their downstream effects. Herein, research has shown that microglia contribute to an accelerated formation of amyloid plaques and tau aggregation [4, 5, 70, 72, 85] which is probably dependent on the activation of the inflammasome [28, 73, 79]. The protective effect of p.P522R in *PLCG2* could modulate the microglial pathways leading to this accelerated pathology. Our results, however, do not support this hypothesis, because we could not find evidence for an association of p.P522R with Abeta_1-42_ pathology or pTau_181_ and tTau in amyloid-negative individuals. Albeit a negative finding, this result should be interpreted with caution, because our AD-focused study design and the limited number of amyloid-negative MCI patients in our sample might have rendered our sample underpowered to detect significant effects of p.P522R on neurodegenerative markers in the absence of amyloidosis. Consequently, additional studies in samples enriched for non-AD dementias are now needed to test our observation.

Interestingly, the stronger effect of p.P552R on tau pathology and cognitive decline in the presence of amyloid pathology offers, in turn, a possible explanation for the lack of association between p.P522R and cognitive decline in population-based studies. The cognitive decline observed in older individuals from the general population is thought to derive from an increased vulnerability of the brain to the initiation of neurodegenerative and non-pathological processes [6, 18, 25, 31]. However, in populations of MCI patients, the prevalence of amyloid and other neuropathologies is increased and exerts a stronger effect on the cognitive decline as compared to cognitively unimpaired participants [26, 32] who form the majority of participants in population-based studies [62]. In fact, MCI cases show a 20–30% higher prevalence of amyloid positivity compared to cognitively normal individuals, independently of age range analyzed [32, 63], as well as a higher prevalence of tau and Lewy body pathology [2]. Our line of arguments receives further support from the observation that the prevalence of amyloid and tau pathology increases with age [7] as well as the probability of being carrier of the p.P522R variant [78]. Thus, it is tempting to speculate that slower cognitive decline in MCI patients and prolonged survival of p.P522R carriers in older ages is due to a modulation of the neuroinflammatory response to progressive pathological protein aggregation, such as amyloid, which leads, in turn, to increased neuronal survival and, finally, improved cognition contributing to longevity [23].

We also examined the potential biological mechanisms underlying the observed effects of p.P522R. Given the compelling evidence supporting the link between TREM2 and APOE at the molecular level, we search for potential shared pathways between *TREM2*, *APOE*, and *PLCG2*. In supporting this hypothesis, the three genes are involved in microglial response within various neurodegenerative conditions. Importantly, APOE and TREM2 might be linked to PLCG2 at the molecular level, as APOE is a ligand of TREM2 [84] and TREM2 is a surface receptor upstream of PLCG2-signaling cascade [54]. Further supporting their functional connection, all three genes modulate similar AD endophenotypes. This includes the effect described here for the APOE-ε4 allele and p.P522R in *PLCG2* on the cognitive decline. In addition, in 2019, a rare protective coding variant in *APOE*, different from the APOE-ε2 allele, was found to mitigate downstream effects of amyloid-beta formation [3], as observed for p.P522R in our study. Furthermore, similar to p.P522R, CSF levels of sTREM2 and the p.R47H variant in TREM2 are both strongly associated with tau pathology [46, 60]. In the case of sTREM2, its CSF levels also show specific alterations in amyloid positive individuals without tau pathology [74]. In line with these molecular and phenotypic links, our unsupervised trans-coregulatory network analysis based on microarray data from multiple tissues and experimental conditions suggested that *APOE*, *PLCG2*, and *TREM2* share a common, general co-expression network. This network contains the AD hub-gene *TYROBP* [88] and it is highly enriched for the complement cascade and genes differentially expressed in microglia from AD patients [49] or mouse models of AD (FXFAD model; [39]) or tauopathies (hMAPT -P301S model; [19]). Thus, several biological processes that have been identified as crucial for the pathogenesis of AD but also other neurodegenerative dementias [14, 21] may not only involve *APOE* and *TREM2,* as previously described [67] but also *PLCG2*. Consequently, taking our present and previous research, it is tempting to speculate that this shared mechanism could include the adaption of microglia to a neurodegenerative environment that may contribute to the protective effect across different neurodegenerative dementias, as hypothesized before. Among the pleiotropy of potential underlying pathways, our co-expression network analysis highlights several mechanisms. Those could involve altered downstream TREM2 signaling including the co-expression network members SPP1 and GPNMB [44, 69]. Alternatively, those mechanisms could include the differential regulation of the signaling of CD14 and Toll-like receptors [86, 87] or complement receptors [9, 48]. Importantly, complement cascade activation is observed in several neurodegenerative diseases, including AD, DLB, and FTD [21, 80] and is strongly involved in synapse loss [27] which is strongly related to cognitive impairments [76]. These candidate pathways require further experimental investigation to establish a role in the mediation of the effect of p.P522R on AD, as well as in other neurodegenerative dementias. In fact, several efforts are currently ongoing to gain insight on the molecular mechanisms of *PLCG2* variant in the immune pathway connecting it with other AD risk genes.

From a therapeutic point of view, PLCG2-directed therapeutic approaches might, therefore, offer a novel opportunity to complement drugs directed at amyloid clearance or production by modulating the downstream effects of amyloid pathology mediated by microglia [12, 28, 51, 71]. Particular interest, herein, deserves the observation that the protective effect of p.P522R can apparently counteract the deleterious effect of APOE-ε4 on cognitive decline for several years and on tau pathology in MCI. Both effects probably derive from the similar effect sizes of the two genetic variants. Of note, APOE-ε4 showed its strongest effect on amyloid pathology and only a smaller but independent effect on tau pathology, suggesting that APOE-ε4 influences AD pathogenesis more upstream in the amyloid cascade than p.P522R. Thus, therapeutic approaches targeting *PLCG2* function might complement those aiming at APOE function. However, our data also suggest that the buffering properties of p.P522R against APOE-ε4-induced deficits seem to decrease as the disease progresses. This reduction in effect may be produced by chronical exposure of microglia to deleterious insult undermining the protective function of microglia including the protective effect given by p.P522R in *PLCG2*. Understanding the pathways underlying the protective effect of *PLCG2* and its buffering properties may help to develop therapeutic targets which can counteract this progressive collapse of the protection delaying or even preventing the beginning of the dementia stage.

### Strength and limitations

A considerable strength of our study is the recruitment and analysis of one of the largest samples of MCI patients with longitudinal follow-up and CSF biomarkers. This data set offers the unique opportunity to study the effect of p.P522R at this disease stage and to define the link between its effects on clinical data and the underlying pathophysiological process. Besides, we used an agnostic co-expression network analysis that does not rely on specific cell or tissue expression profile experiments that are usually based in very low numbers and, therefore, prone to measurement error. Importantly, using a general approach relying on a wide range of experiments led us to replicate the previous findings on expression network [10, 68, 88] and also led us to the discovery of a novel links between *APOE* and *TREM2*–*PLCG2* signaling. Furthermore, the integrative analysis of biomarker, cognitive, and gene-expression data provides the most comprehensive description of the role of PLCG2 in AD pathogenesis to date.

However, our study has also limitations. Our results are based on a limited number of carriers due to the low frequency of the p.P522R variant. Especially when examining the interaction of p.P522R with APOE-ε4 or Aβ_1-42_, the limited number of carriers might have rendered our study underpowered for quantifying the exact effect size of the interaction. Thus, investigations of independent samples are now necessary to confirm these observations. Nevertheless, the consistency of the effect of p.P522R across cohorts and phenotypes supports the robustness of our findings. Additionally, amyloid and tau burden from cognitively healthy individuals were not available. Since the trajectory of amyloid pathology is expected to reach a plateau in late disease stages [30], the lack of cognitively normal patients with CSF samples might have limited our ability to detect the effects of p.P522R on CSF-Aβ_1-42_ levels. We were, however, able to detect an effect for the APOE-ε4 allele. Finally, confirmation of findings from the co-regulatory network analysis is still required using microglia expression data.

In conclusion, our data link the protective effect of p.P522R in *PLCG2* to lower CSF concentrations of pTau_181_ and tTau and slower cognitive decline in MCI patients, particularly in amyloid positive individuals and with an effect size similar to that of *APOE*. We present converging evidence, suggesting that the rare variant p.P522R in *PLCG2* might reduce the effect of amyloidosis upon tau pathology and cognitive decline. Therapies targeting the druggable enzyme PLCG2 [17, 68] might, thus, provide a novel therapeutic approach with the potential for disease modification in AD might as well buffer the deleterious effect of APOE-ε4.

## Electronic supplementary material

Below is the link to the electronic supplementary material.

Supplementary file1 (PDF 1791 kb)
