# Supplementary material for: PLCG2 protective variant p.P522R modulates tau pathology and disease progression in patients with mild cognitive impairment
Source: Acta Neuropathol. 2020 Mar 12;139(6):1025–44. doi: 10.1007/s00401-020-02138-6 (PMC7244617; doi:10.1007/s00401-020-02138-6)
Supplement: Supplementary file 1 — Supplementary file1 (PDF 1791 kb) [file 401_2020_2138_MOESM1_ESM.pdf]

Supplementary text 1. Samples description

German Dementia Competence Network cohort (DCN)

The DCN cohort includes 1,095 patients with mild cognitive impairment (MCI) and 648 cases with mild Alzheimer's disease (AD) clinical dementia syndrome who were recruited from 14 university hospital memory clinics across Germany between 2003 and 2005 [23]. Exclusion criteria were substance abuse or dependence, insufficient German language skills, multi-morbidity, comorbid condition with excess mortality, circumstances that would have made regular attendance at follow-up visits questionable and lack of an informant.

An MCI diagnosis was assigned following the Winblad criteria [60]. The criteria were operationalized as having complaints of cognitive deficit in daily living and objectified decline of cognitive abilities (more than 1 SD), as evidenced by age-corrected standardized tests, in at least 1 of the following domains: verbal learning and memory, nonverbal learning and memory, word fluency, naming, visuoconstruction, cognitive speed or executive function. Minor impairments in complex activities of daily living (i.e. a Bayer-ADL (B-ADL) score <4) were accepted [15]. The diagnosis of dementia of the Alzheimer's type was assigned according to NINCDS-ADRDA criteria [35].

All patients underwent extensive neuropsychological testing at baseline and at a maximum of three annual follow-up visits with a maximal follow-up time of 4.1 years. MRI and CSF samples were optionally acquired. Valid phospholipase-C-γ2 (PLCG2) genotypes were available for 766 patients. Of those, 348 provided CSF data. We excluded 6 patients due to age below 50 resulting in a sample of 342 samples. From the 537 MCI patients who provided data on longitudinal cognition, 8 patients had to be excluded because of missing covariates (7 due to missing APOE-ε4 genotype and one patient due to missing information on education). Therefore, 529 MCI patients were included in the cognitive decline analyses. Of these, 252 were also included in the analysis of CSF biomarkers.

The study was approved by the respective ethics committees, and written informed consent was obtained from all participants before inclusion.

Interdisciplinary Memory Clinic at the University Hospital of Bonn (UH Bonn)

Patients from the memory clinic at the university hospital of Bonn were recruited with a similar protocol as in the DCN and received a diagnosis of MCI according to Winblad criteria [60]. For the diagnosis of dementia of the Alzheimer's type, diagnoses were assigned according to the NINCDS-ADRDA criteria [35] and based on clinical history, physical examination, neuropsychological testing (using the CERAD neuropsychological battery, including the MMSE), laboratory assessments, and brain imaging. Out of 91 patients with CSF data and PLCG2 genotypes available, 4 had to be excluded due to age below 50 resulting in 87 eligible patients. The majority of the CSF samples were quantified in the laboratory in Erlangen (n=60) while the remaining 27 samples were quantified in the laboratory in Bonn.

The study was approved by the respective ethics committees, and written informed consent was obtained from all participants before inclusion.

Amsterdam Dementia cohort (ADC)

The sample consisted of 502 MCI patients who visited the memory clinic of the Alzheimer Center of the VU University Medical Center (VUmc) between 1996 and 2016 [55]. Inclusion and exclusion criteria of the Amsterdam Dementia Cohorts are described elsewhere [55]. Patients underwent an extensive standardized assessment, including a physical and neurologic examination, medical history based on informant, neuropsychological assessment, laboratory tests, CSF investigation and magnetic resonance investigation (MRI) of the brain.

MCI diagnoses were made independent of the CSF results in a consensus meeting attended by neurologists, neuropsychologists, and nurses. Petersen's criteria were used until the beginning of 2012 when the National Institute on Aging–Alzheimer's Association (NIA-AA) criteria for MCI were implemented [1, 42]. The diagnosis of dementia of the Alzheimer's type was made according to the NINCDS-ADRDA criteria [35].

Follow-up was conducted on an annual basis in a standardized fashion. Up to 12 follow-up visits were performed and the maximum follow-up time was 11.6 years. Among 384 patients with CSF data and valid PLCG2 genotypes, 3 were excluded due to age below 50.

The medical ethics committee of the VU University Medical Center approved the study protocol for the use of clinical data for research purposes and the biobank protocol for storage and use of DNA. Written informed consent was obtained from all individuals before inclusion.

Fundació ACE (FACE)

The FACE sample comprised MCI patients that were recruited from the Diagnostic Unit of FACE in Barcelona, Spain between 1996 and 2015. Recruitment, inclusion and exclusion criteria and assignment of a diagnosis of MCI in the ACE dataset were described in detail previously [12]. All MCI patients were assessed with the neuropsychological battery of Fundació ACE (NBACE), the Mini-Mental State Examination (MMSE), the Hachinski Ischemia Scale, the BDRS and the Neuropsychiatric Inventory Questionnaire (NPI-Q) [5, 6, 13]. The MMSE was measured at all visits. A diagnosis of MCI was assigned according to the Petersen criteria [42] and the classification of Lopez et al [12, 26, 27]. All subjects had a CDR [37] of 0.5, were autonomous at the time of enrolment but did not report deficits in general intellectual abilities. Minor impairments in complex ADL were allowed. All MCI diagnoses were made by neurologists, neuropsychologists, and social workers at a consensus conference.

Follow-up assessments were conducted on an approximately annual basis. Up to 20 follow-up visits with a maximal observation time of 18.3 years were conducted. From the total sample of 1,095 MCI patients with p.P522R genotype, 3 patients were excluded due to missing APOE-ε4 information leaving 1092 patients for the analysis of cognitive decline.

The study was approved by the respective ethics committees, and written informed consent was obtained from all participants before inclusion. The study protocol complied with national legislation and the Code of Ethical Principles for Medical Research Involving Human Subjects of the World Medical Association.

Alzheimer's Disease Neuroimaging Initiative (ADNI)

Data used in the preparation of this article were obtained from the Alzheimer's Disease Neuroimaging Initiative (ADNI) database (adni.loni.usc.edu). The ADNI was launched in 2003 as a public-private partnership, led by Principal Investigator Michael W. Weiner, MD. The primary goal of ADNI has been to test whether serial magnetic resonance imaging (MRI), positron emission tomography (PET), other biological markers, and clinical and neuropsychological assessment can be combined to measure the progression of mild cognitive impairment (MCI) and early Alzheimer's disease (AD). For up-to-date information, see www.adni-info.org. For the current study, patients with early and late MCI from the ADNI1, ADNI GO and ANDI2 cohorts were included. Late MCI was operationalized as a MMSE between 24 and 30, self- or informant reported complaints about worsening memory performance, a CDR rating of 0.5, objective education-adjusted memory impairment as measured by the Wechsler Memory scale Logical Memory II test but absence of impairment in other cognitive domains and absence of dementia or essential impairments in activities of daily living. For early MCI subjects, less stringent criteria for the definition of objective impairment in memory were applied (0.5-1.5 SD below the mean of cognitively normal individuals). In addition, all participants had to be between 55 and 90 years of age, fluent in English or Spanish and have a Hachinski score<=4 and willing to undergo ADNI test procedures and longitudinal follow-up. The diagnosis of mild DAT was assigned according to NINCDS/ADRDA criteria for probable AD [35].

After the baseline visit, patients were assessed every 3 month and after one completed year, every 12 month. Assessments included detailed neuropsychological testing and clinical assessment as well as biomarker measurements including the collection of CSF samples used in our analyses. For the current analysis, we used only participants reporting to be non-hispanic whites to reduce the risk of population stratification in this sample.

Written informed consent was obtained for participation in these studies, as approved by the institutional review board at each participating center.

The German study on aging, cognition and dementia cohort (AgeCoDe)

The AgeCoDe study is a general practice (GP) registry-based longitudinal study in elderly individuals that recruited patients aged 75 years and above in six German cities from 2003 to 2004 [29]. Exclusion criteria were consultations only by home visits by the GP, residence in a nursing home, severe illness the GP would deem fatal within 3 months, insufficient facility in German, deafness or blindness, lacking the ability to consent and not being a regular patient of the participating practice. A total of 3,327 patients gave informed consent for participation and received follow-up assessments every 18 months. Up to 8 follow-up visits were considered with a maximal follow-up time of 12.3 years. All assessments were performed by trained physicians and psychologists at the patient's home environment using standardized questionnaires. Valid PLCG2 genotype data were available in 1,978 participants. Of those, 1,967 participants were all included in the analysis of the cognitive decline in the population-based studies. Nine participants were excluded due to missing APOE-ε4 genotype and two participants were excluded due to age below 75 indicating failure to meet the AgeCoDe inclusion criteria.

At baseline and each follow-up, AgeCoDe participants received an MCI diagnosis according to Winblad criteria [60] which were operationally defined by evidence for cognitive impairment (-1SD) in the neuropsychological test battery of the SIDAM, self-report of deterioration of memory functions and preserved activities of daily living (SIDAM-ADL-Scale) [63]. Using the data from the baseline assessment of the full sample, the prevalence of MCI was estimated at 15.4% [29] which closely resembles meta-analytic prevalence estimates form general population-based studies [48]. For the analysis of the cognitive decline in MCI patients, 929 participants who received an MCI diagnosis at any visit of the AgeCoDe cohort were included. However, for this analysis, we used the time point of MCI diagnosis as the new analysis baseline, i.e. no observations recorded before the MCI diagnoses were considered. This approach was chosen to align the data from AgeCoDe MCI patients with the data from the other three memory clinic cohorts with longitudinal data (i.e. DCN, ADC, and FACE) since the observation also started at the time point of MCI diagnosis in these cohorts. For the MCI analysis up to 8 follow-up visits were included with an observation time of up to 12.2 years.

The present study was approved by the respective ethics committees, and written informed consent was obtained from all participants before inclusion. All study procedures complied with national legislation and the Code of Ethical Principles for Medical Research Involving Human Subjects of the World Medical Association.

Three City study (3C)

The 3C study enrolled 9,294 non-institutionalized participants aged 65 years or above, sampled from the electoral rolls of three French cities of Bordeaux, Dijon, and Montpellier between 1999 and 2001 [2]. Inclusion criteria were (1) living in these cities or their suburbs and registered on the electoral rolls, (2) aged 65 years and over and (3) not institutionalized.

Each participant signed informed consent. Partial refusal of the participation in specific parts of the examination (e.g. blood sampling or magnetic resonance imaging) did not result in exclusion from the study. Details about the study design of 3C were reported previously [2]. Health-related data were collected using standardized questionnaires during face-to-face interviews. Participants were assessed for up to 10 years in approximately 2 years intervals.

The 3C study did not implement a standardized, regular assessment of MCI diagnosis at each visit but an assessment of dementia diagnosis. 2.2% of the 3C study participants were classified as demented at baseline [2].

The study protocol was approved by the ethical committee of the University Hospital of Kremlin-Bicêtre. The study was conducted according to the principles expressed in the Declaration of Helsinki.

Longitudinal Aging Study Amsterdam (LASA)

The prospective cohort study Longitudinal Aging Study Amsterdam (LASA) consists of a nationally representative sample of older adults aged between 55 and 85 years who were recruited from the regions around Zwolle, Oss, and Amsterdam in the Netherlands. The LASA study incorporates three cohorts. The first LASA cohort included 3,107 participants and was selected from the municipal registries of the regions of the Netherlands named above between 1991 and 1992, with an oversampling of the oldest old and older men. The subjects of the first LASA cohort were followed-up for up to 24 years in 3-year intervals. A second cohort was

recruited between 2002 and 2003 in the same regions as the original cohort and consisted of 1,002 subjects that were assessed for up to 13 years again in three-year intervals. The third cohort included 1,023 participants that were recruited between 2012 and 2013 and received two assessments (in three-year intervals) so far. At all visits, interviews were performed by trained interviewers in the respondents' home environment. Further information is provided by Huisman and colleagues [18]. In LASA, no formal assessment of dementia or MCI was performed. In total 5,132 participants were included at baseline (3,107 participants were included in cohort 1, 1,002 in cohort 2 and 1,023 in cohort 3). The LASA study is conducted in line with the Declaration of Helsinki and was approved by the medical ethics committee of the VU University medical center.

Supplementary text 2. Genotyping

To genotype the rs72824905 variant (p.P522R) in samples from FACE, UKB, DCN and AgeCoDe, a custom TaqMan® SNP genotyping assay was designed using the available Applied Biosystems online assay design tool. Oligonucleotide primers were ordered from Applied Biosystems (Thermo Fisher Scientific) and performed according to the manufacturer's instructions in a Quantstudio-6™ Real-Time PCR Systems (Thermo Fisher Scientific). The quality of the assay was confirmed by direct sequencing of heterozygous samples. Assays' accuracy was checked by including positive and negative controls in each experiment.

ADC was genotyped using the Illumina Global Screening Array (Infinium-global-screening-array-24-v1 with GSAsharedCUSTOM_20018389_A2) and applied established quality control methods [8]. We used high-quality genotyping in all individuals (individual call rate > 98%, variant call rate > 98%), individuals with sex mismatches were excluded and Hardy–Weinberg equilibrium-departure was considered significant at $p < 1 \times 10^{-6}$. The PLCG2 variant was part of the custom content of the GSA array.

In ADNI, genotyping was performed using the Illumina Human610-Quad BeadChip, HumanOmniExpress BeadChip and Illumina Omni 2.5M platforms as previously described and applied established quality control methods were applied including exclusion of duplicates, highly related individuals and non-caucasian subjects (based on a ±6 SD from the man cut-off for the first principal component and a ±3 SD cut-off for the second principal component). The p.P522R genotypes were derived from imputation using the HRC Michigan Imputation Server [34]. At the HRC server, SHAPEIT was used to phase the data, and imputation was performed using the HRC reference panel (hrc.r1.1.2016) and a cut-off for imputation quality of $R^2=0.3$. Imputed genotypes were returned by the service. For the current analysis, we used "best-guess" genotypes of p.P522R based on the platform with the highest quality (genotype probability>=0.8).

In the 3C study, genomic DNA samples of 6,636 individuals were transferred to the French Centre National de Génotypage (CNG) as part of a previous replication effort [52]. Samples that passed DNA quality control were genotyped using the Agena Bioscience MassARRAY platform. We also excluded samples with more than three missing genotypes and males heterozygous for X chromosome variants present within the panel. Variants were excluded based on missingness >5%, Hardy–Weinberg equilibrium (in cases and controls separately) < 1 × 10 −5, and differential missingness between cases and controls < 1 × 10 −5. After exclusion, 6,201 samples were available.

The LASA cohorts were genotyped using commercial genotyping arrays and then followed a genotyping quality control and an imputation step. For genotyping, the Infinium-global-screening-array-24-v1 was used in 1,899 samples and the AXIOM-NL array was utilized for 625 [11]. The following quality control (QC) criteria were used with the individuals genotyped: individuals with a call rate <98%, an excess heterozygosity rate or a gender mismatch, and individuals which were duplicates or PCA outliers using a PCA projection of the study samples onto 1KG were removed. A total of 2,358 samples passed the QC (1,779 GSA and 579 Axiom array). At the variant level following QC criteria were used: all monomorphic markers and markers with Hardy-Weinberg $p>10^{-6}$ or call rate <98% were removed. For imputation, the dataset was prepared using scripts provided online (Haplotype Reference Consortium [HRC] imputation preparation and checking, version 4.2.5). Imputation to the HRC was performed at the HRC Michigan Imputation Server [34]. At the HRC server, SHAPEIT2 (version 2, .r790) was used to phase the data, and imputation was performed using the HRC reference panel (version 1.0) using Minimac 3. Imputed genotypes were returned by the service. Imputation quality (R2) was 0.89 for GSA and 0.93 for the Axiom assay. They were combined in the analysis due to highly similar MAF of 1.38% and 1.02% in the axiom and GSA assay, respectively.

Supplementary figure 1A-G. Plots of the first two principal components of the population structure per cohort

Supplementary figure 1a. DCN cohort

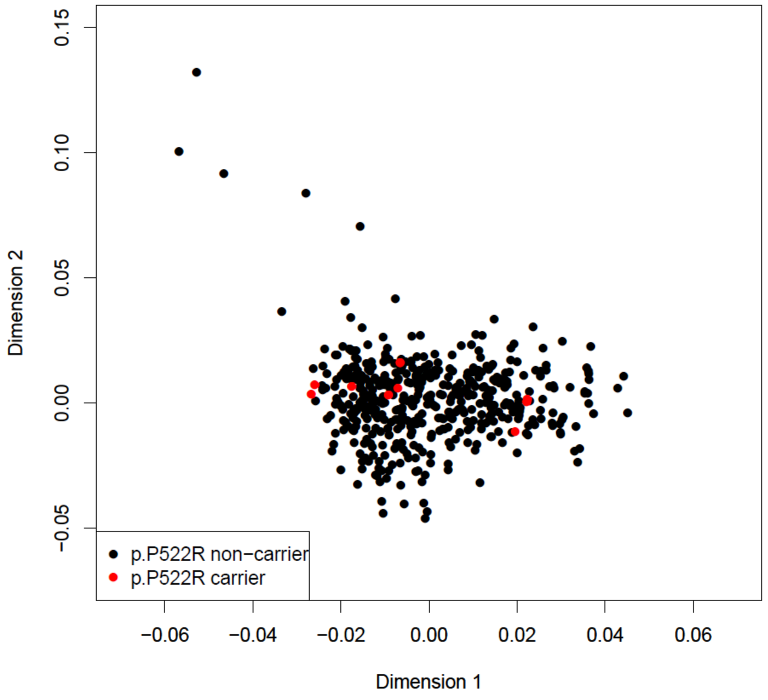

Supplementary figure 1b. ADC cohort

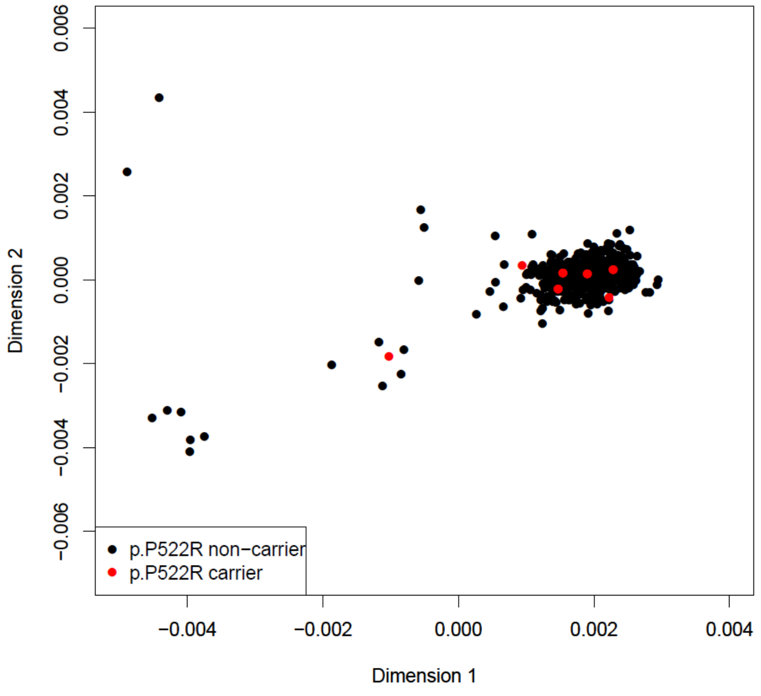

Supplementary figure 1c. FACE cohort

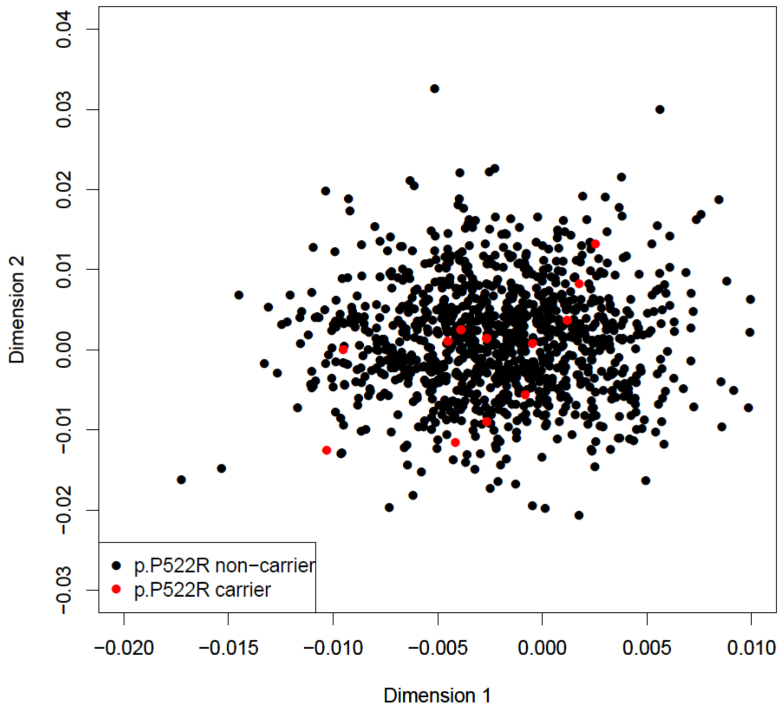

Supplementary figure 1d. ADNI cohort

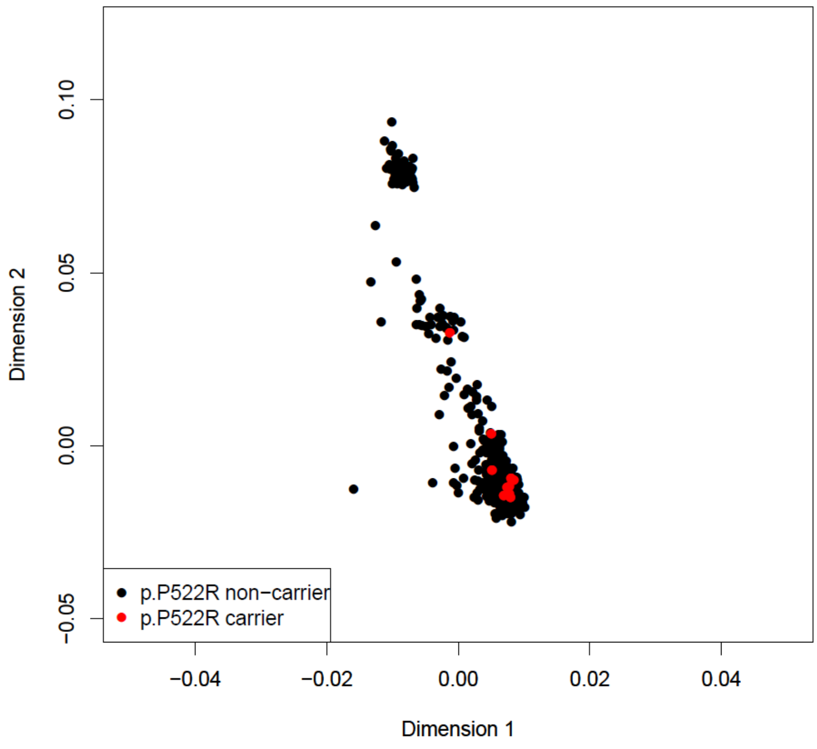

Supplementary figure 1e. AgeCoDe cohort

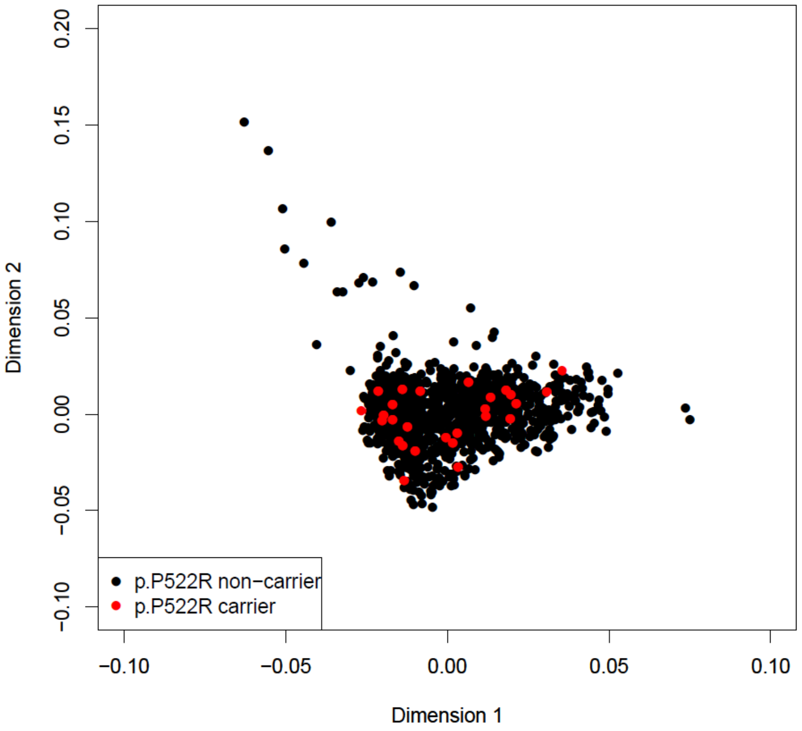

Supplementary figure 1f. 3C cohort

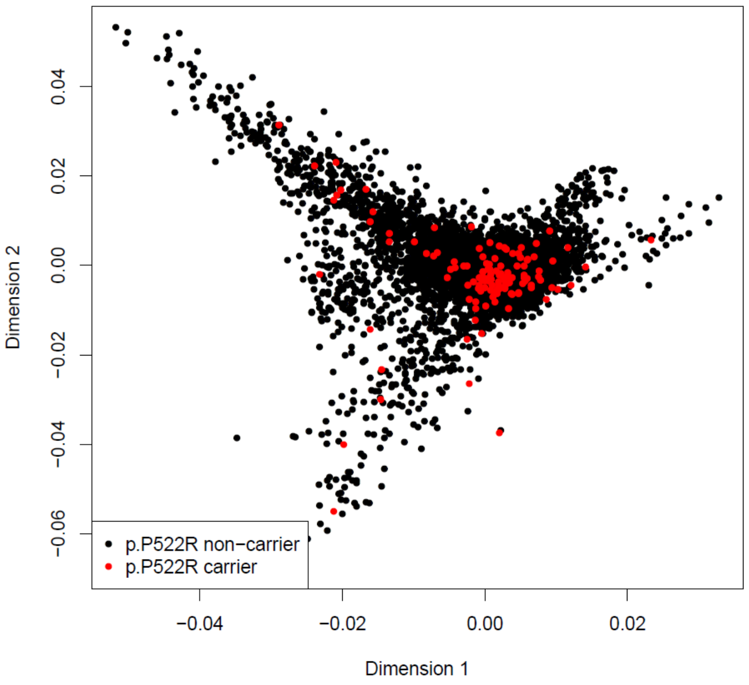

Supplementary figure 1g. LASA cohort

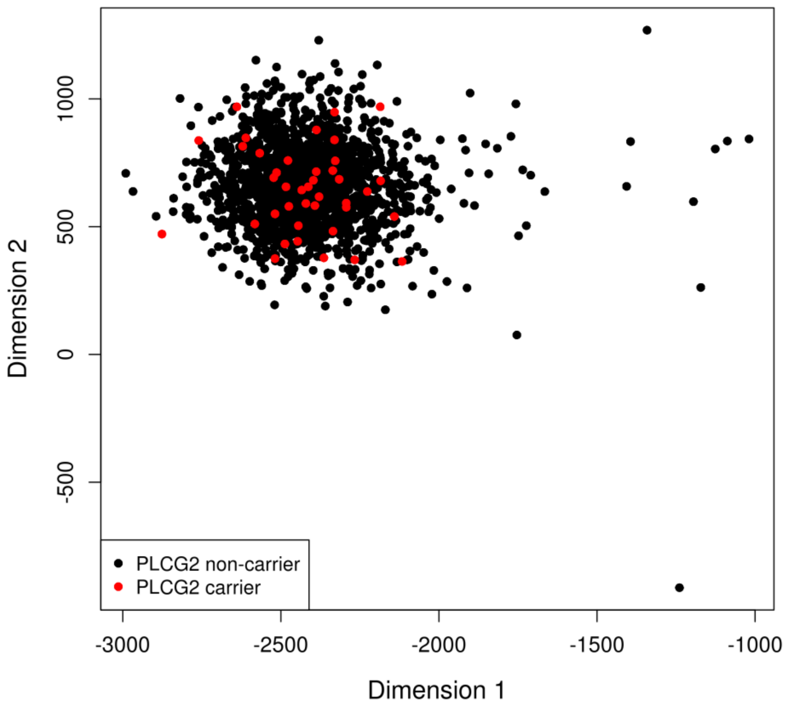

Supplementary text 3. Examination of the effect of population stratification

To evaluate the impact of adjusting for potential population stratification using principal components (PC), we restricted the analysis of the effect of p.P522R in MCI to those samples with GWAS data available. Thus, we could calculate PC for the sample and include them in the analysis. All cases from ADNI and ADC had GWAS data available, while 632 (68.2%) MCI patients of AgeCoDe and 348 (65.8%) DCN and 1,060 (97.1%) of the FACE cohort provided GWAS data. We then rerun only analyses showing significant associations in the main analyses in the paper with and without inclusion of the first two PCs in our model. We assessed the perceptual change in the parameters. As suggested previously on confounding effects [31], changes of less than 10% were considered an indication that the effects found in our study are independent of adjustments for PCs. Data was not pooled across cohort as PCs were computed per cohort and therefore represent a cohort-specific measure to correct for population stratification that could not be harmonized across cohorts.

In the analyses of cognitive decline, we did not re-analyze the data from ADC since the low number of carriers and the short follow-up for these carriers had already affected the stability of our estimations in the main analyses (see supplementary figure 2).

Cohort	FACE							AgeCoDe						
Statistical model	Not adjusted for PCs			Adjusted for first two PCs				Not adjusted for PCs			Adjusted for first two PCs			
	χ^2	df	p	χ^2	df	p		χ^2	df	p	χ^2	df	p	
Joint effect of p.P522R on cognitive change	3.958	2	0.138	3.923	2	0.141		3.649	2	0.161	3.617	2	0.164	
	Est	SE	p	Est	SE	p	%Δ	Est	SE	p	Est	SE	p	%Δ
P.P522R	1.096	0.701	0.118	1.062	0.697	0.128	-3.09	0.074	0.383	0.848	0.084	0.382	0.825	14.72
P.P522R*time	0.410	0.216	0.058	0.419	0.217	0.053	2.16	0.167	0.133	0.211	0.167	0.132	0.206	0.30
P.P522R*timeQ	-0.018	0.034	0.601	-0.024	0.035	0.481	34.81	0.003	0.017	0.855	0.00279	0.01668	0.867	-9.12
PC1				0.227	0.072	0.002					0.015	0.036	0.679	
PC1*time				0.008	0.028	0.779					0.014	0.013	0.285	
PC1*timeQ				-0.001	0.004	0.840					-0.002	0.002	0.478	
PC2				-0.226	0.148	0.127					0.010	0.035	0.769	
PC2*time				-0.002	0.062	0.980					0.000	0.013	0.976	
PC2*timeQ				-0.007	0.007	0.326					0.001	0.002	0.727	
Cohort	DCN							ADNI						
Statistical model	Not adjusted for PCs			Adjusted for first two PCs				Not adjusted for PCs			Adjusted for first two PCs			
	χ^2	df	p	χ^2	df	p		χ^2	df	p	χ^2	df	p	
Joint effect of p.P522R on cognitive change	0.847	2	0.655	0.907	2	0.635		1.588	2	0,452	1.905	2	0.386	
	Est	SE	p	Est	SE	p	%Δ	Est	SE	p	Est	SE	p	%Δ
P.P522R	0.042	0.631	0.947	-0.041	0.615	0.946	-199.14	0.395	0.581	0.496	0.435	0.579	0.453	9.20
P.P522R*time	0.338	0.373	0.366	0.333	0.366	0.363	-1.52	0.181	0.172	0.293	0.196	0.171	0.252	8.29
P.P522R*timeQ	0.161	0.223	0.472	0.179	0.219	0.415	11.26	-0.002	0.035	0.946	-0.002	0.036	0.949	-0.00
PC1				0.171	0.058	0.003					0.023	0.658	0.972	
PC1*time				-0.015	0.038	0.692					-5.368	10.454	0.608	
PC1*timeQ				-0.054	0.022	0.012					-0.586	3.123	0.851	
PC2				-0.011	0.052	0.831					5.752	2.792	0.039	
PC2*time				-0.045	0.031	0.143					1.213	2.024	0.549	
PC2*timeQ				0.003	0.018	0.862					-0.047	0.569	0.934	

Results indicated that there were no changes in the estimates of the effect of p.P522R on the linear decline over time. For the quadratic term, we observed a stronger change with PC inclusion. However, the effect of p.P522R

in the main analysis and in all individual cohorts derived from the interaction of p.P522R and the linear term indicating that the stronger change for the quadratic term could represent random noise. In line with this, the p-values for the association of p.P522R with the cognitive decline that takes into account both, the linear and the quadratic term, remained unchanged by PC adjustment. We therefore conclude that PC adjustment had no effect on the associations of p.P522R in each cohort.

For the analyses on CSF biomarkers, no change above 10% in the estimates for the effect of p.P522R was observed for $pTau_{181}$and tTau for which we observed a significant influence in our main analysis. Changes in estimates for $A\beta_{1-42}$ may again arise from random noise in the non-significant association with p.P522R. Of note, the effect observed on $A\beta_{1-42}$ in DCN is in the opposite direction as in the ADNI cohort suggesting again a random noise more than a true association.

Outcome	Variable	DCN							ADNI						
		Not adjusted for PCs			Adjusted for first two PCs				Not adjusted for PCs			Adjusted for first two PCs			
		Est	SE	p	Est	SE	p	%Δ	Est	SE	p	Est	SE	p	%Δ
$A\beta_{1-42}$	P.P522R	-0.096	0.078	0.217	-0.079	0.073	0.279	-17.55	0.017	0.068	0.802	0.024	0.068	0.721	42.53
	PC1				2.962	0.732	0.000					-3.614	5.250	0.492	
	PC2				1.894	0.769	0.014					0.154	0.981	0.876	
$pTau_{181}$	P.P522R	-0.167	0.082	0.044	-0.160	0.081	0.048	-3.97	-0.102	0.077	0.184	-0.109	0.077	0.156	6.46
	PC1				-0.502	0.827	0.545					4.660	5.810	0.423	
	PC2				2.077	0.927	0.026					0.060	1.086	0.956	
tTau	P.P522R	-0.150	0.096	0.118	-0.153	0.095	0.107	1.68	-0.131	0.082	0.110	-0.137	0.082	0.094	4.71
	PC1				-1.914	0.969	0.049					2.361	6.288	0.708	
	PC2				1.104	1.094	0.314					-0.581	1.174	0.621	

Supplementary text 4. CSF collection and harmonization

Supplementary text 4.1: CSF collection

In the ADC cohort, a lumbar puncture was performed using a 25G needle. The CSF was collected in polypropylene tubes and centrifuged at 1800/2100 *g* for 10 min at 4°C. The CSF was immediately stored at −20°C until further analysis (maximum 2 months). CSF levels of $A\beta_{1\text{–}42}$ and $pTau_{181}$ and total Tau were measured using commercial ELISA immunoassays. Quantification was performed in the neurological laboratory of the VU University Medical Center in Amsterdam following the protocols described by Mulder and colleagues [39]. For the definition of AD biomarker abnormality, we applied previously published cut-off values, which are the standard in the Amsterdam CSF laboratory [39, 66]. Abnormal CSF- $A\beta_{1\text{–}42}$ was defined as values <640 pg/ml, abnormal CSF-total tau (tTau) was defined as values >375 pg/ml, and CSF- $pTau_{181}$ >52 pg/ml.

In the DCN and the memory clinic Bonn samples, lumbar punctures from the L3/L4 or L4/L5 intervertebral region were performed at the respective department of neurology or psychiatry. The CSF samples were kept on ice for a maximum of 1 h and then centrifuged for 10 min (2000 *g* at 4°C). Samples were aliquoted to 0.25 ml and stored in polypropylene tubes at −80 °C until analysis. All CSF samples from DCN and 257 samples from the memory clinic of Bonn were sent to the Department of Psychiatry in Erlangen for quantification. Levels of $A\beta_{1\text{–}42}$ and $pTau_{181}$ and total Tau were measured using commercially available ELISA immunoassays INNOTEST® β-amyloid$_{(1\text{–}42)}$ and INNOTEST® PhosphoTAU$_{(181p)}$ (Innogenetics), in accordance with the protocols described by Lewczuck and colleagues in an ISO9001–certified laboratory under a routine quality-control regimen (intra-assay coefficients of variation: 2.3%–5.9%; interassay coefficients of variation: 9.8%–13.7%) [24, 25]. We performed all analyses in duplicate and used the mean of the two. We defined abnormally low CSF $A\beta_{1\text{–}42}$ < 600 pg/mL, abnormally high CSF tTau > 300 pg/mL, and abnormally high CSF $pTau_{181}$ > 60 pg/mL based on DCN specific, previously published cutoff values.

In 27 CSF samples collected at the university hospital Bonn, CSF samples were processed using the protocol established by the local biomarkers laboratory. Briefly, CSF samples kept at 4 °C until processing for biobank storage at -80 °C. Processing was completed on the day of lumbar puncture and samples were stored for no longer than 4 weeks until analysis. Samples and calibrators were run in duplicates, and samples with a coefficient of variation (CV) > 20% were repeated. A pooled and aliquoted CSF sample was run as an internal standard on each assay plate to control for inter-run variance. For AD core biomarkers, the V-PLEX Aβ Peptide Panel 1 (6E10) Kit (K15200E), the V-PLEX Human Total Tau Kit (K151LAE) (Mesoscale Diagnostics LLC, Rockville, MD, USA), as well as the INNOTEST PHOSPHO TAU(181P) kit (81581, Fujirebio, Ghent, Belgium) was used. Laboratory specific cut-off values to define abnormal CSF levels were $A\beta_{1\text{–}42}$ < 350 pg/mL, tTau > 470 pg/mL, $pTau_{181}$> 57 pg/mL.

In the ADNI cohort, CSF was collected as previously described [50]. In brief, CSF samples were collected in the morning after an overnight fast. After collection and transfer into polypropylene tubes, CSF samples were frozen on dry ice within 1 hour after collection followed by shipping to the ADNI Biomarker Core Laboratory at the University of Pennsylvania Medical Center on dry ice. Afterward, thawing (1 hour) at room temperature and gentle mixing, aliquots (0.5 mL) were prepared and stored in bar code–labeled polypropylene vials at −80°C. All CSF biomarkers were quantified using the multiplex xMAP Luminex platform (Luminex Corp, Austin, TX) with Innogenetics (INNO-BIA AlzBio3; Ghent, Belgium; for research use-only reagents) immunoassay kit–based reagents. We used < 192 pg/mL as the cut-off for $A\beta_{1\text{–}42}$, >23 pg/mL as the cut-off for $pTau_{181}$ and 93 pg/mL as the cut-off for tTau as previously recommended.

Supplementary text 4.2: Harmonization of batch effects

Due to technical differences, CSF samples analyzed with different quantification batches are not measured on the same scale. To pool and jointly analyze CSF samples quantified with different batches, a harmonization procedure is required. In this study, the method described by Zhou and colleagues was applied [65]. This method allows the harmonization of non-overlapping samples. To do so a transformation is determined that converts CSF samples analyzed with different batches to the same scale and provides a p-value to assess the accuracy of the transformation. The Matlab (MATLAB Release 2016b The MathWorks, Inc., Natick, Massachusetts, United States) implementation created by Zhou and colleagues was used (https://github.com/hzhoustat/PNAS2018). For the harmonization efforts, we included patients with dementia of the Alzheimer's type in the analyses to broaden the range of pathologies and the sample size. This might improve the removal of methodological differences.

To ensure that the transformation only harmonizes differences in the scales induced by batches it is necessary to account for sample differences due to sample selection bias or different population characteristics. To this end, a set of relevant covariates have to be identified whose influence is then removed by stratifying the sample according to these covariates. To identify those covariates, graphical cause model techniques can be applied. Zhou and colleagues demonstrated that for the harmonization of CSF in AD research, age and diagnosis are important determinants. However, especially in the context of genetics and samples with highly different age ranges, the APOE-ε4 allele is another important factor since it influences CSF levels and the age at onset of Alzheimer's disease [28, 41]. Hence, APOE-ε4 was added to the causal graph proposed by Zhou and colleagues [65]. A graphical representation of the extended graph is presented below:

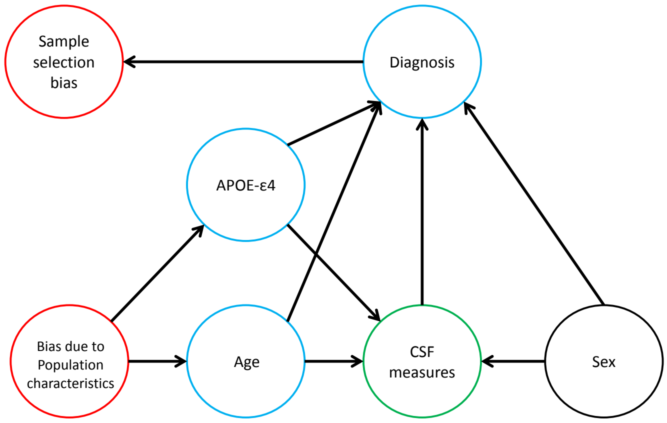

As can be seen from the figure, bias induced by sample selection and population characteristics is selectively linked to the CSF measures via the three variables age, APOE-ε4 and diagnosis. Removing their influence will enable correction for differences in scales between different CSF batches.

On the other hand, no differences in population characteristics between the samples from the DCN and UKB cohort were expected, regardless of the laboratory and the batch used for quantification since patients referring to the memory clinic of the university hospital of Bonn were included in both cohorts and since inclusion criteria for both cohorts were largely similar. Both cohorts did indeed not differ in frequency of age groups (i.e. <60/60-70/71-75/>75), $\chi^2(3)=2.19$, $p=0.534$), APOE-ε4 ($\chi^2(1)=2.80$, $p=0.094$) and gender ($\chi^2(1)=2.37$, $p=0.124$). However, the frequency of diagnoses (i.e. MCI or dementia) was different and the diagnosis was therefore used for stratification during the computation of the harmonization transformation.

MCI patients of the DCN and the ADC cohort are expected to differ concerning relevant sample characteristics because they were recruited in different countries using different study protocols. Both cohorts differed concerning age ($\chi^2(3)=14.140$, $p=0.002$) and APOE-ε4 ($\chi^2(1)=8.36$, $p=0.004$) but not with regard to gender ($\chi^2(1)=2.37$, $p=0.124$). The former two variables were used to stratify the samples during the harmonization.

For the ADNI cohort, sample characteristics were also expected to differ from those of the CSF Erlangen sample. In line with this expectation, we observed significant differences regarding age ($\chi^2(3)=101.5$, $p<0.001$) and diagnosis ($\chi^2(1)=81.7$, $p<0.001$). Unexpectedly, both samples showed additional differences in their gender distribution ($\chi^2(1)=11.3$, $p=0.001$) but not in the APOE-ε4 frequency ($\chi^2(1)=0.4$, $p=0.843$). To avoid bias in the transformation process due to difference and gender and over-stratification resulting in too small samples for harmonization by conditioning on APOE-ε4, we used age, gender and diagnosis to stratify the samples for harmonization.

To harmonize the samples, we applied a linear transformation to the samples quantified in the laboratory in Bonn or Amsterdam to harmonize them with the samples processed in Erlangen (DCN and the majority of the samples from the UHB cohort) which remained untransformed. Non-significant p-values indicated that the transformations were appropriate for $A\beta_{1\text{–}42}$ ($p=0.354$), $pTau_{181}$ ($p=0.763$) and total tau ($p=0.454$) processed in Bonn as well as for $A\beta_{1\text{–}42}$ ($p=0.524$), $pTau_{181}$ ($p=0.511$) and total tau ($p=0.713$) samples processed in Amsterdam. Similar results were obtained for $A\beta_{1\text{–}42}$ ($p=0.940$), $pTau_{181}$ ($p=0.908$) and total tau ($p=0.872$) samples in the ADNI cohort.

Supplementary text 5. Neuropsychological assessments

In all MCI samples, the MMSE was considered for the assessment of cognition [13]. The test is a global screening for dementia consisting of 30 items. This test was chosen because it was the only test available across all cohorts that provided a sensitive global assessment of cognitive change across different cognitive domains. Previous research has shown that it provides good sensitivity to cognitive changes in MCI patients [46].

In the 3C study, the global cognition of the participants was also assessed using the MMSE. Besides, the Isaac set test (IST) [20] and the Benton visual retention test (BVRT) [4] were assigned as a measure of verbal fluency and episodic memory, respectively. In the 3C study, the IST requires the naming of words of different, consecutively administered semantic categories (i.e. colors, animals, fruits and cities) for 15 seconds. The BVRT consists of 15 stimulus cards presented for 10 seconds. Afterward, the participants are asked to select each stimulus among 3 distractors.

In AgeCoDe, the CERAD delayed word list recall and animal fluency task of the CERAD neuropsychological test battery [38] were considered for cognitive assessments in addition to the MMSE. Similar to the 3C study, they assess verbal fluency, episodic memory, and global cognition, respectively. The CERAD delayed word list recall consists of 10 words that are presented and have to be recalled in three consecutive learning trials. After a delay, the participant is asked to freely recall from long-term memory. The animal fluency task requires the participant to name as many animals as possible in one minute.

In LASA, the global cognition of the participants was also assessed using the MMSE. Episodic memory was assessed using the 15 Words Test, the Dutch version of the Auditory Verbal Learning Test [47, 49]. The tests consisted of 15 words which were learned during 3 trials. After every trial, the respondent was asked to recall as many words as possible. After a distraction period of 20 minutes, the respondent was asked to name again the learned words. The total number of words learned during 3 tests is the recall score (range 0-45). The number of words reproduced after 20 minutes is the delayed recall score (range 0-15) which was used in this research [9].

Supplementary Table 1. Software and gene expression databases used in the analysis

Software	Web address	Analysis
R package LCMM [45]	https://cran.r-project.org/package=lcmm	Analysis of cognitive decline
R package robustbase [32]	http://CRAN.R-project.org/package=robustbase	Analysis of continuous CSF biomarkers
R package nnet [58]	https://cran.r-project.org/web/packages/nnet/index.html	Multinominal regression
R package mgcv [62]	https://cran.r-project.org/web/packages/mgcv/index.html	Analysis of continuous CSF biomarkers using generalized additive models
R package itsadug [57]	https://cran.r-project.org/web/packages/itsadug/index.html	Posterior simulation of generalized additive models
CSF batch harmonization software [65]	https://github.com/hzhoustat/PNAS2018	Harmonization of batch effects
Mplus [40]	https://www.statmodel.com/	Structural equation modeling
GeneFriends [54]	http://www.genefriends.org/	Generation of trans co-expression networks for APOE, TREM2, and PLCG2
WebGestalt [64]	http://www.webgestalt.org/option.php	Analysis of enrichment of biological processes in gene sets
STRING [53]	https://string-db.org/	Analysis of Protein-Protein Interactions in the co-expression network shared by APOE, PLCG2, and TREM2
R package homologene	https://cran.r-project.org/web/packages/homologene/index.html	Enrichment for genes differentially expressed in microglia

Supplementary text 6. Statistical methods

Supplementary text 6.1: Latent process linear mixed models for the analysis of the cognitive decline

We chose to model the continuous cognitive decline in patients with MCI rather than conversion to dementia since the analysis of continuous traits can improve statistical power to detect genetic determinants [51]. Besides, simulation has demonstrated that the application of linear mixed models of cognitive decline may outperform survival analysis concerning the sensitivity for modulators of disease progression [10].

However, cognitive tests used as outcomes in these analyses frequently show unequal interval scaling and ignoring this issue can introduce bias in the analysis of cognitive decline [44]. Taking this source of bias into account is recommended by current guidelines for longitudinal dementia research [59]. Therefore, we used linear mixed models with a latent process as implemented in the R package LCMM [45]. These models jointly estimate a latent process representing the true change of cognition over time and a link function that relates this process to the observed cognitive measurements. To select the most appropriate link function adjusting for unequal interval scaling, we compared several options: a linear link function, a beta link function, and quadratic I-splines with varying numbers of knots placed at the percentiles or equidistant along with the distribution of the outcome. All possible link functions were fitted using models including a random intercept and a linear or quadratic polynomial trend of time in the random and/or fixed effects (i.e. linear fixed and random effect, quadratic fixed and linear random effect, and quadratic random and fixed effect). An evaluation of the need for non-linear decline is recommended by guidelines for statistical analysis in dementia research [59]. Besides, the omission of a relevant non-linear effect from the mixed model can induce spurious effects for predictors that are correlated with the person-specific mean of the variable showing the non-linear effect that was omitted [3]. In our model, omitting a non-linear trend of time would possibly introduce spurious effects for p.P522R because the variant is related to survival [56] so that p.P522R can be expected to show a higher mean observation time.

Only polynomial trends up to the second-order (i.e. quadratic) were examined. More complex models including higher polynomial terms of time could not be reliably fitted in the limited number of p.P522R carriers in our cohorts and would have resulted in overfitting to random variation in the longitudinal cognitive data of the carriers. In the pooled cohort of MCI patients, we allowed the fixed effects of the polynomial trends of the time to vary across cohorts. All models were also combined with different zero-mean Gaussian stochastic processes that take into account correlations between the observations besides the random effects. We considered an uncorrelated errors structure, a first-order autoregressive and Brownian motion process.

Among all fitted models, those with the lowest BIC and appropriate model fit according to a visual inspection of residual plots were chosen to assess the effect of p.P522R. These factors (i.e. link function, the shape of the cognitive decline trajectory, residual error structure) were evaluated in combination since the interaction of these factors concerning model fit is hard to predict a priori.

The procedures described above were repeated for each outcome in each of the population-based cohorts (AgeCoDe, 3C, and LASA) and the sample of MCI patients pooled across cohorts. However, for the analysis of the MMSE in LASA and the 3C study, a beta-link function was chosen a priori since this link function has been shown to provide a good modulation of the unequal interval scaling of this test in the literature and provided the best model fit in the AgeCoDe cohort and the MCI sample [44]. The selection of the same link function in the analyses may also contribute to the comparability of results across cohorts.

Of note, the time scales used to set up the linear mixed model with a latent process differed between the MCI samples and the population-based cohorts. In the population-based cohorts, chronological age at each visit was used as the most natural time scale to study the cognitive change in elderly populations [59]. We also included age at baseline to model the convergence of age-related cognitive trajectories of individuals from different birth cohorts [16, 59]. Analyses, therefore, focus on the age-related cognitive change over time.

In the MCI samples, however, time from diagnosis was used because for this analysis, the focus was more on disease progression in the at-risk stage for AD. Nevertheless, we controlled for age at baseline to take into account different chronological ages of the MCI patients and to correct for birth cohort differences. All MCI samples were pooled and analyzed jointly. This approach is called integrative data analysis and it is recommended in case of rare predictor variables to enable the application of more complex, accurate statistical models, reduce the influence of outlying observations and to maximize the power to detect associations [7]. The use of the MMSE as a common test across cohorts assured that the cognitive decline was assessed using a homogenous, harmonized outcome measure in all cohorts.

Only after the selection of the best fitting combination of link-function, the shape of the trajectory of the cognitive decline and additional correlation structure between observations, covariates and p.P522R were included in the model as well as their interaction with all polynomials of time. The significance of the effect of p.P522R on the cognitive decline was assessed using a multivariate Wald test of the joint effect of all rare variant-time interactions as implemented in the LCMM package.

Supplementary text 6.2: Computation of effect sizes in linear mixed models with non-linear effects

In case of a non-linear trajectory of cognition over time, the effect of a predictor under study (i.e. p.P522R or APOE-ε4 in this analysis) changes may change over time as the speed of cognitive changes is not constant over the observation period. It is therefore not straightforward to provide a single effect size of the association of the predictor and the cognitive decline. Consequently, we computed effect sizes at multiple time points of the cognitive trajectory to evaluate the possible changes in the association. To this end, we derived expected differences in cognition at each time point concerning the baseline level of cognition based on the fixed effect estimates in the latent process linear mixed models. As those estimates were on an arbitrary, latent scale, we used the expected variance of the latent process at the last evaluated tie point to standardize the estimates. Estimates of this variance were derived from the random effect variance-covariance matrix of the latent process linear mixed models.

Supplementary text 6.3: Generalized additive models

Generalized additive models are a flexible method to assess the relationship between a dependent variable and a set of independent variables where the functional form of the relationship does not have to be linear. To this end, it is assumed that the dependent variable is related to the independent variables via a smooth function. The model can be written as:

$$\mathrm{E}(Y) = \mathrm{a} + \mathrm{s}_1(\mathrm{x}_1) + \mathrm{s}_2(\mathrm{x}_2) + \ldots + \mathrm{s}_\mathrm{n}(\mathrm{x}_\mathrm{n})$$

where Y is the dependent variable, E() is the expected value, a is the intercept of the model, $s_n()$ are smooth functions and $x_1, \ldots, x_n$ are independent variables. The smooth functions can be estimated from the data while taking into account the model complexity and fit to the data. In the current analyses, the R-package mgcv [62] was used to estimate the generalized additive models. The smooth functions were represented by thin plate regression splines [61].

To assess the interplay of p.P522R and $A\beta_{1\text{–}42}$ levels in CSF concerning tTau and $pTau_{181}$, we estimated a varying-coefficient model that estimated a smooth relationship between $A\beta_{1\text{–}42}$ levels and tTau or $pTau_{181}$ levels as well as an effect for p.P522R that was allowed to vary smoothly with the levels of amyloid pathology:

$$\mathrm{E}(tTau) = \mathrm{a} + \mathrm{s}_1(\mathrm{A}\beta_{1-42}) + \mathrm{s}_2(\mathrm{A}\beta_{1-42}) \cdot \text{p.P522R} + s_3(age) + \text{sex} + \text{APOE-}\varepsilon 4 + \text{CSF sample}$$

Analyses were controlled for sex, APOE-ε4 and CSF sample as parametric linear effects. For age, a smooth term was estimated to take into account a potential non-linear relationship to CF biomarkers.

To infer those levels of $A\beta_{1\text{–}42}$ at which p.P522R is associated with tTau or $pTau_{181}$ levels, we performed posterior simulations as implemented in the "plot_smooth" and "plot_diff" functions form the R-package itsadug [57]. Estimates of the expected relationship of $A\beta_{1\text{–}42}$ and tTau or $pTau_{181}$ for p.P522R carrier and non-carrier as well as expected differences between the two groups were derived. Simultaneous confidence intervals (as opposed to point-wise confidence intervals) were computed to account for multiple testing.

Supplementary text 6.4: Structural Equation modeling

We fitted structural equation models to assess mediation of the effect of p.P522R on the cognitive decline via CSF biomarkers in Mplus version 7.31 [40]. As recommended we did structural equation modeling instead of estimates from separate regression and linear mixed models to estimate mediation since this can slightly increase the efficiency of the estimation and simplifies the use of multiple mediators and the modeling of their interplay [19]. To assess the fit of all structural equation models, we provide the root mean square error of approximation (RMSEA), the comparative fit index (CFI) and the standardized root mean square residual (SRMR). An RMSEA<0.5, a CFI>0.95 and an SRMR<0.08 indicate a good fit to the data [17].

We modeled the effect of p.P522R on $A\beta_{1\text{–}42}$ and $pTau_{181}$ taking into account the effect of $A\beta_{1\text{–}42}$ on $pTau_{181}$ that is postulated by the amyloid cascade hypothesis [21]. All three variables were allowed to predict the cognitive decline that was derived using latent growth curve models. In the second series of models, $pTau_{181}$ was replaced

by tTau. We did not include tTau and $pTau_{181}$ in a single model due to the high correlation between the two biomarkers in our sample (r=0.82).

Since the structural equation model does not allow a straightforward implementation of link functions taking into account unequal interval scaling in cognitive tests as implemented in the latent process linear mixed models described above, the normalized version of the MMSE was used [43]. This version of the MMSE is corrected for unequal interval scaling due to a transformation derived from several large cohort studies.

The latent growth curve models used to model the cognitive decline in the normalized MMSE require data to be assessed at fixed time points that should not show large inter-individual variation and reasonable overlap of observations at different time points for the same individuals (covariance coverage) to result in a successful estimation. We, therefore, included data that were assessed in yearly intervals for up to 4 years which reflects a common follow-up scheme across cohorts. One additional assessment after 6 months was included due to sufficient covariance coverage.

All estimations were performed using full information maximum likelihood (FIML) that can handle missing data which is missing at random. We modeled the cognitive decline using three latent factors representing cognitive level at baseline, linear and quadratic change over time, respectively. The need for a quadratic slope was assessed using the Akaike information criterion (AIC) and sample-size adjusted BIC.

Indirect effects were calculated as the product of the effect of p.P522R on a CSF biomarker and the effect on the cognitive change from baseline. Due to the use of a linear and quadratic change of the normalized MMSE over time, we assessed the indirect effects at different time points across the observed trajectory (i.e. the change from baseline after 1 year, 2 years, 3 years and 4 years). For the indirect effect of $pTau_{181}$ and tTau we used the following formula:

Indirect effect = $a(b_1L_s+b_2L_q)$

a=effect of p.P522R on CSF $pTau_{181}$ or tTau
b_1=effect of CSF biomarker on the linear slope of cognitive change over time
b_2= effect of CSF biomarker on quadratic of cognitive change over time
L_s=loading of the linear slope on the time point of interest
L_q=loading of the quadratic slope on the time point of interest

For $A\beta_{1\text{–}42,}$ we also considered the effect of this biomarker on $pTau_{181}$ and tTau, respectively, as mentioned above. Therefore we applied the formula:

Indirect effect = $a_{11}(b_{11}L_s+b_{12}L_q)+a_{21}c(b_{21}L_s+b_{22}L_q)$

a_{11}=effect of p.P522R on $A\beta_{1\text{–}42}$
a_{21}=effect of p.P522R on $pTau_{181}$ or tTau
b_{11}=effect of $A\beta_{1\text{–}42}$ on the linear slope of cognitive change over time
b_{21}=effect of $pTau_{181}$ or tTau on the linear slope of cognitive change over time
b_{12}= effect of $A\beta_{1\text{–}42}$ on quadratic of cognitive change over time
b_{22}= effect of $pTau_{181}$ or tTau on quadratic of cognitive change over time
c=effect of $A\beta_{1\text{–}42}$ on $pTau_{181}$ or tTau
L_s=loading of the linear slope on the time point of interest
L_q=loading of the quadratic slope on the time point of interest

Finally, 95%-Confidence intervals for the indirect effects were derived using 1000 bootstrap samples [30]. When examining the interaction effect of p.P522R and CSF biomarkers significance of the interaction was assessed using a multivariate Wald test on the effect of the interaction on the linear and the quadratic slope.

Supplementary text 6.5: Selection of covariates

When assessing the influence of p.P552R, we considered age, gender, education, APOE-ε4 status and cohort as covariates. Education was operationalized as participation in secondary education. APOE-ε4 status was defined as the presence or absence of the APOE ε4 allele.

We chose the covariates to exclude the possibility that a random association between these standard demographic variables and p.P522R might bias our results. Likewise, APOE-ε4 as the strongest genetic risk factor for AD might influence our results due to a random co-occurrence of p.P522R and APOE-ε4. Since none of these variables is thought to mediate the effect of p.P522R on the outcomes under study (i.e. CSF AD biomarkers and cognitive decline) inclusion for these variables might increase the statistical power to detect associations [36]. Cohort was included as a covariate to control for residual differences between samples that were not removed by harmonization strategies (i.e. common neuropsychological test across cohorts, statistical harmonization of continuous CSF AD biomarkers; [65]). In the CSF analyses, CSF sample was used instead of recruiting cohort as a covariate since batch effects are expected to be the most important source of heterogeneity between samples.

In some analyses, additional covariates were included. In the analysis of the cognitive decline in the 3C study, study center was included as an additional fixed effect to control for the difference between the study centers as it is common practice in analyses of the 3C study. In LASA, the platform used for genotyping was included to control for differences between assessment methods of p.P522R. This was not necessary for other cohorts because here, identical genotyping procedures were applied.

Importantly, in the case of the detection of significant associations of p.P522R, analyses were repeated without adjustment for covariates to examine the sensitivity of the results to covariate selection.

Supplementary text 6.6: Enrichment analyses for the gene co-expression network

Fisher's exact test were applied to assess enrichments for genes co-expressed with *PLCG2* and other gene lists. We assumed a total population size of 19,080 genes since according to the number of all genes examined by the GeneFriends tool [54].

To assess the enrichment of PLCG2-related genes co-expressed with *APOE* or *TREM2*, respectively, we used Fisher's exact test (two-sided). When computing these tests, *APOE*, *TREM2*, and *PLCG2* themselves were not considered as a part of the population because those genes were chosen to construct the co-expression network.

To assess the enrichment of differentially expressed genes in microglia under neurodegenerative conditions among genes in the gene set shared between *APOE*, *TREM2*, and *PLCG2*, we first derived lists of those differentially expressed genes from the literature. From the work of Keren-Shaul and colleagues [22], 500 mouse genes differentially expressed in disease-associated compared to homeostatic microglia (see supplementary table 1 of the publication by Keren-Shaul and colleagues [22]) were merged to their human homologs using the homologene package in R and the genes included in the GeneFriends database. This resulted in a list of 320 genes. From the work of Friedman and colleagues [14], we extracted human genes differentially expressed in microglia of wild type compared to hMAPT -P301S mice (see supplementary data 2 of the publication by Friedman and colleagues [14]) were extracted. Of those 318 were included in the GeneFriends database. Regarding differential expressions in human AD patients, we selected the list of the genes published by Mathys and colleagues [33] (see supplementary table 7 of the publication by Mathys et al. 2019 [33]) yielding 75 genes that were also included in the GeneFriends database. For all analyses on differentially expressed genes in microglia under neurodegenerative conditions, we considered *APOE*, *TREM2*, and *PLCG2* as part of the underlying population of genes.

Supplementary Table 2. Estimated association of p.P522R in *PLCG2* and the APOE-ε4 allele with the cognitive decline in the Mini-Mental State Examination derived from the pooled analysis of the MCI cohorts

	Main analysis [a]			Shortened follow-up interval (<=6 years) [a]			Unadjusted analysis		
	Est/ χ^2	SE/df	p	Est/ χ^2	SE/df	p	Est/ χ^2	SE/df	p
Effect of p.P522R in PLCG2 on the cognitive decline									
p.P522R [b]	0.132	0.270	0.626	0.217	0.281	0.441	0.333	0.301	0.268
p.P522R*time	0.186	0.090	0.038	0.222	0.089	0.012	0.250	0.094	0.008
p.P522R*time^2	0.005	0.012	0.653	-0.004	0.031	0.906	-0.002	0.013	0.860
Multivariate Wald test	7.83	2	0.020	6.58	2	0.037	8.95	2	0.011
Effect of APOE-ε4 on the cognitive decline									
APOE-ε4 [b]	-0.749	0.076	$3.96*10^{-23}$	-0.747	0.077	$3.01*10^{-21}$			
APOE-ε4*time	-0.259	0.026	$1.38*10^{-23}$	-0.252	0.025	$5.62*10^{-23}$			
APOE-ε4*time^2	-0.007	0.004	0.124	-0.0003	0.009	0.972			
Multivariate Wald Test	138.33	2	$9.27*10^{-37}$	100.18	2	$1.77*10^{-22}$			

***Note*.** Sample size n=3595. Unequal interval scaling was adjusted using a beta-link function. A Brownian motion process zero-mean Gaussian stochastic process was used to model the correlation between observations besides the random effects. Multivariate Wald test refers to the joint test of the interaction of the genotype (i.e. p.P522R or APOE-ε4) with polynomials of time.
a: Analysis adjusted for cohort, age at baseline, sex, education, and APOE-ε4 status.
b: effect on cognitive function at the median of observation time (i.e. two years).
Est=Estimate; SE=Standard error; p=p-value; df=degrees of freedom; χ^2=statistic of the multivariate Wald test.

Supplementary Figure 2. Cohort-specific effects of p.P522R on the cognitive decline in the Mini-Mental State Examination

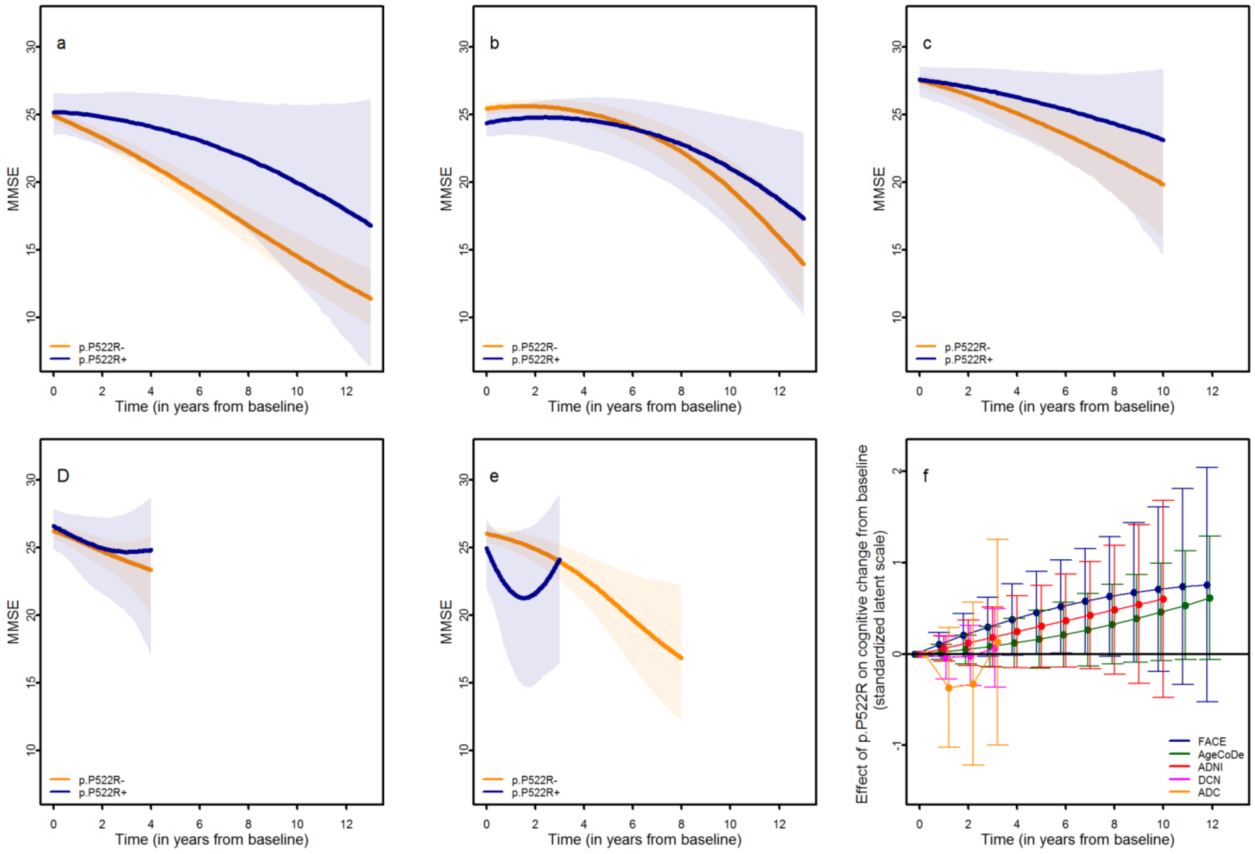

Note. **a:** Fundacio ACE (FACE), **b:** German study on aging, cognition, and dementia (AgeCoDe), **c:** Alzheimer's disease neuroimaging **(**ADNI) cohort, **d:** Dementia competence network (DCN) cohort **e:** Amsterdam Dementia cohort (ADC). In the ADC cohort, only one carrier provided more than 1 year of follow-up (and up to 4 years of follow-up) so that the displayed trajectory relies almost completely on one observation and is therefore prone to overfitting and measurement error. **f:** Difference between p.P522R carrier and non-carrier on the cognitive change from baseline. Differences were derived based on the p.P522R*time and p.P522R*time² terms of the linear mixed model with a latent process using the pooled sample of all cohorts but an interaction effect between p.P522R and polynomials of time and cohort. Differences are displayed on the scale of the latent process that was standardized using the expected variance of the latent process of the last time point considered (i.e. 12 years after baseline, see supplementary text 6.2). The prediction was not derived from stratified analyses to avoid different scaling of the latent process across cohorts due to the difference in the estimated link function relating the latent process to the observed MMSE values.

Supplementary Table 3. Estimated association of p.P522R in PLCG2 and APOE-ε4 with the cognitive decline in the Mini-Mental State Examination derived from the analysis of each MCI sample separately

	FACE[a]			AgeCoDe[b]			DCN[c]			ADC[d]			ADNI[e]		
	**Est/ χ^2**	**SE/ df**	**p**	**Est/ χ^2**	**SE/ df**	**p**	**Est/ χ^2**	**SE/ df**	**p**	**Est/ χ^2**	**SE/ df**	**p**	**Est/ χ^2**	**SE/ df**	**p**
**Effect of p.P522R in PLCG2 on the cognitive decline**															
p.P522R [f]	1.108	0.705	0.116	-0.518	0.361	0.152	0.084	0.654	0.897	-1.579	1.314	0.230	0.395	0.581	0.496
p.P522R*time	0.414	0.217	0.056	0.073	0.124	0.557	0.123	0.324	0.705	0.922	0.846	0.276	0.181	0.172	0.293
p.P522R*time^2	-0.019	0.035	0.584	0.010	0.016	0.499	0.094	0.186	0.614	0.712	0.517	0.168	-0.002	0.035	0.946
Multivariate Wald test	3.991	2	0.136	2.079	2	0.354	0.265	2	0.876	1.905	2	0.386	1.588	2	0.452
**Effect of APOE-ε4 on the cognitive decline**															
APOE-ε4 [f]	-0.745	0.155	$1.42*10^{-6}$	-0.445	0.145	0.002	-0.484	0.195	0.013	-0.367	0.156	0.018	-1.272	0.154	$1.64*10^{-16}$
APOE-ε4*time	-0.306	0.047	$9.29*10^{-11}$	-0.199	0.052	0.00013	-0.061	0.117	0.599	-0.084	0.067	0.219	-0.340	0.049	$2.58*10^{-12}$
APOE-ε4*time^2	-0.004	0.008	0.566	-0.011	0.008	0.192	0.033	0.062	0.596	0.005	0.024	0.812	0.0001	0.010	0.993
Multivariate Wald Test	59.346	2	$1.39*10^{-13}$	27.907	2	$8.71*10^{-7}$	1.817	2	0.403	1.726	2	0.421	62.162	2	$3.17*10^{-14}$

Note**.** The analyses were adjusted for cohort, age at baseline, sex, education and APOE-ε4 status. Unequal interval scaling was adjusted using a beta-link function. A Brownian motion process zero-mean Gaussian stochastic process was used to model correlation between observations besides the random effects. Multivariate Wald test refers to the joint test of the interaction of the genotype (i.e. p.P522R or APOE-ε4) with polynomials of time.

a: sample size n=1092.
b: sample size n=927.
c: sample size n=529.
d: sample size n=431.
e: sample size n=616.
f: effect on cognitive function at the median of observation time (i.e. two years).
Est=Estimate; SE=Standard error; p=p-value; df=degrees of freedom; χ^2=statistic of the multivariate Wald test.

Supplementary Table 4. Results from a linear mixed model with a latent process using the pooled sample of MCI patients and including the interaction between p.P522R and APOE-ε4

	**Est**	**SE**	**p**
APOE-ε4	-0.751	0.076	$6.08*10^{-23}$
p.P522R	0.134	0.325	0.679
APOE-ε4*p.P522R	0.029	0.479	0.952
APOE-ε4*time	-0.262	0.026	$8.56*10^{-24}$
p.P522R *time	0.120	0.110	0.28
APOE-ε4*p.P522R*time	0.185	0.175	0.290
APOE-ε4*time^2	-0.006	0.004	0.208
p.P522R*time^2	0.019	0.014	0.193
APOE-ε4*p.P522R*time^2	-0.043	0.026	0.094
**Multivariate Wald Test**	**χ^2**	**df**	**p**
Interaction APOE-ε4* p.P522R*polynomials of time	2.87	2	0.238

Note: Est=Estimate; SE=Standard error; p=p-value; df=degrees of freedom; χ^2=statistic of the multivariate Wald test;

Supplementary Table 5. Associations of p.P522R in PLCG2 with cognitive decline in multiple neuropsychological domains in population-based samples

3 City Study	MMSE[a] (n=5870)			Episodic memory (Benton test. n=5837)[b]			Verbal fluency (Isaac Set Test. n=5858)[d]		
	Est/ χ^2	SE/df	p	Est/ χ^2	SE/df	p	Est/ χ^2	SE/df	p
p.P522R	0.026	0.117	0.821	0.078	0.10	0.416	0.117	0.145	0.418
p.P522R*time	-0.014	0.017	0.400	-0.003	0.01	0.804	0.012	0.019	0.523
p.P522R*time^2	0.0002	0.002	0.904	-0.0002	0.002	0.915	0.002	0.0004	0.848
Multivariate Wald Test	0.710	2	0.701	0.106	2	0.948	0.534	2	0.766
**LASA**	**MMSE[a] (n=2216)**			**Episodic memory (15-WT test. n=2213)[a]**					
	Est/ χ^2	SE/df	p	Est/ χ^2	SE/df	p			
p.P522R	-0.098	0.160	0.540	-0.105	0.204	0.607			
p.P522R*time	0.003	0.013	0.802	0.019	0.017	0.254			
p.P522R*time^2	-0.001	0.001	0.371	-	-	-			
Multivariate Wald Test	0.989	2	0.610	-	-	-			
**AgeCoDe**	**MMSE[a] (n=1965)**			**Episodic memory (CERAD DR. n=1961)[b]**			**Verbal fluency (CERAD animal fluency. n=1969)[c]**		
	Est/ χ^2	SE/df	p	Est/ χ^2	SE/df	p	Est/ χ^2	SE/df	p
p.P522R	0.105	0.223	0.639	-0.350	0.324	0.281	-0.273	0.297	0.358
p.P522R*time	0.035	0.035	0.311	-0.032	0.041	0.434	-0.015	0.038	0.699
p.P522R*time^2	-0.002	0.005	0.718	0.002	0.006	0.713	-0.005	0.006	0.356
Multivariate Wald Test	1.217	2	0.544	0.740	2	0.691	0.90	2	0.636

Note: Est=Estimate; SE=Standard error; p=p-value; df=degrees of freedom; χ^2=statistic of the multivariate Wald test; LASA=Longitudinal aging study Amsterdam; AgeCoDe= German study on aging, cognition and dementia; MMSE=Mini Mental State Examination; CERAD=Consortium to Establish a Registry for Alzheimer's Disease neuropsychological test battery; DR=delayed recall; 15-WT test=Dutch version of the Auditory Verbal Learning Test (15-word test).

[a] unequal interval scaling of the outcome was adjusted using a beta-link function.

[b] unequal interval scaling of the outcome was adjusted using a I-spline function with 6 equidistant interior knots.

[c] unequal interval scaling of the outcome was adjusted using a I-spline function with 4 interior knots placed at the quartiles of the outcome distribution.

[d] unequal interval scaling of the outcome was adjusted using a I-spline function with 3 equidistant interior knots.

In the 3C study and AgeCoDe, a Brownian motion process residual error structure was used. In LASA, a first-order autoregressive residual error structure was applied. Multivariate Wald test refers to the joint significance test of the interaction of the genotype (i.e. p.P522R or APOE-ε4) with the polynomials of time.

Supplementary figure 3. Effect of p.P552R in PLCG2 on the cognitive decline in the Three-City study (3C)

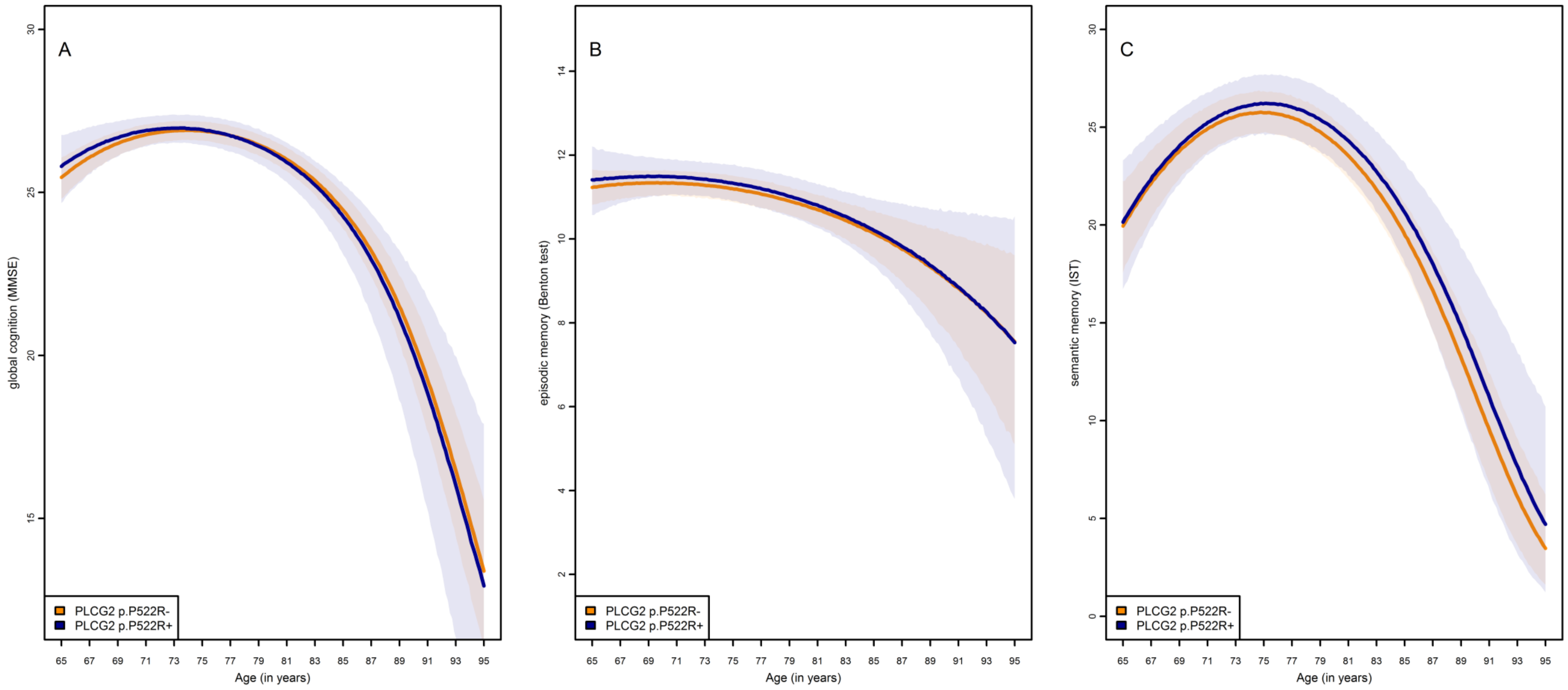

*Note***.** A) Effect of cognitive decline in global cognitive function measured by the Mini-Mental State Examination.
B) Effect of cognitive decline in episodic memory measured by the Benton test.
C) Effect of cognitive decline in semantic memory measured by the Isaac Set test.

Supplementary figure 4. Effect of p.P552R in PLCG2 on the cognitive decline in the Longitudinal Ageing Study Amsterdam (LASA)

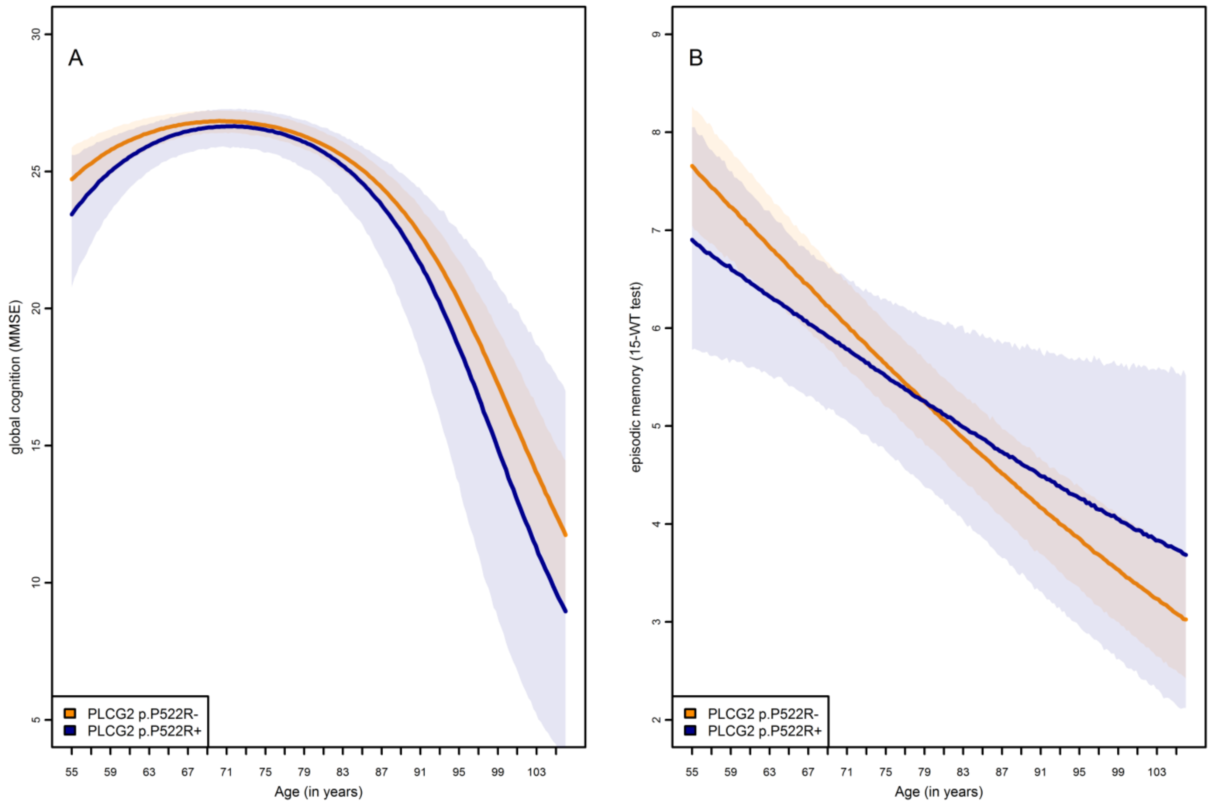

*Note***.** A) Effect of cognitive decline in global cognitive function measured by the Mini-Mental State Examination.
B) Effect of cognitive decline in episodic memory measured by the delayed recall measure of the Auditory Verbal Learning Test.

Supplementary figure 5. Effect of p.P552R in PLCG2 on the cognitive decline in the AgeCoDe cohort

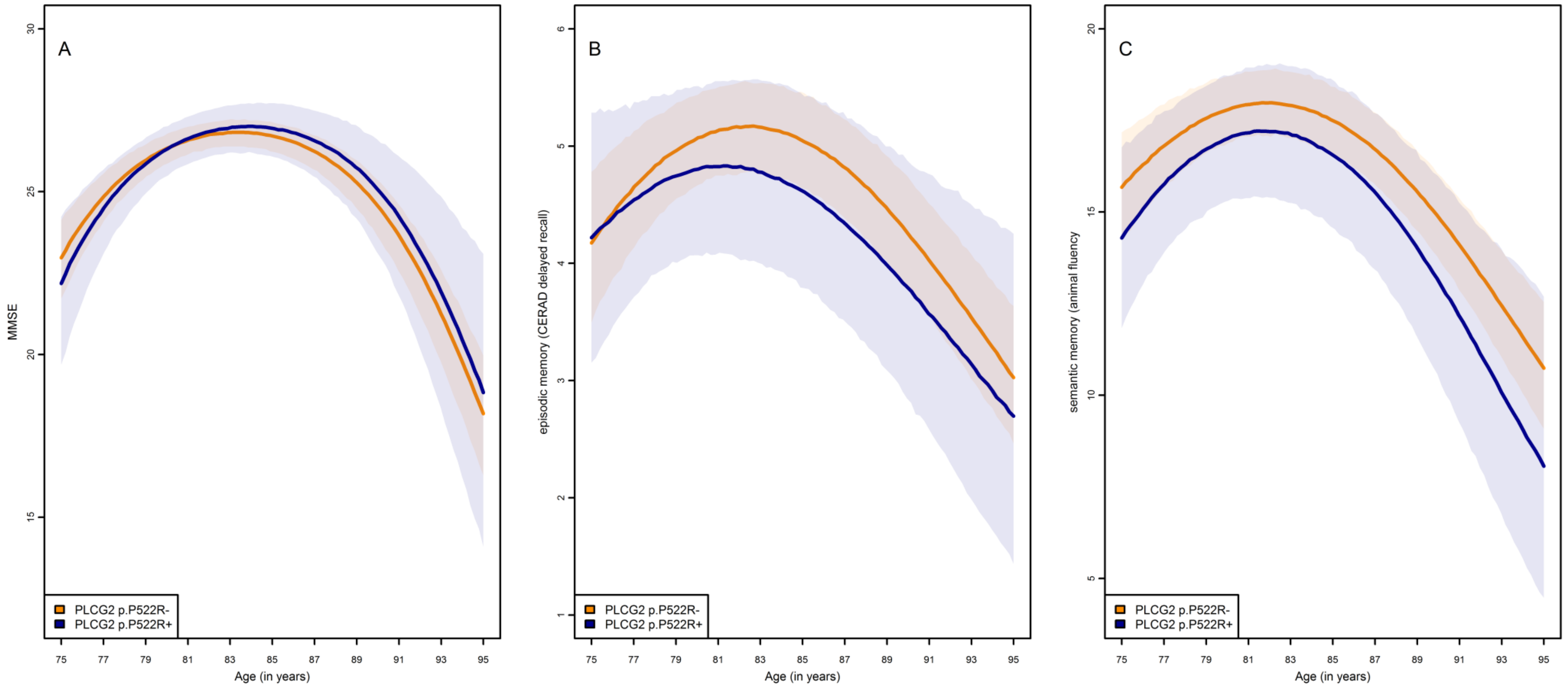

Note. A) Effect of cognitive decline in global cognitive function measured by the Mini-Mental State Examination.
B) Effect of cognitive decline in episodic memory measured by CERAD word list delayed recall.
C) Effect of cognitive decline in semantic memory measured by CERAD verbal fluency task.

Supplementary Table 6. Associations of p.P522R with CF levels of AD biomarkers

Type of analysis	$A\beta_{1-42}$ levels				$pTau_{181}$ levels				tTau levels			
	N (p.P522R)	Est. (SE)	p	d (95%-CI)	N (p.P522R)	Est. (SE)	p	d (95%-CI)	N (p.P522R)	Est. (SE)	p	d (95%-CI)
**European MCI patients**												
main	11	-0.06 (0.06)	0.273	-0.33 (-0.93 to 0.26)	11	-0.12 (0.06)	0.068	-0.56 (-1.16 to 0.04)	11	-0.11 (0.07)	0.113	-0.48 (-1.08 to 0.11)
unadjusted	11	-0.09 (0.07)	0.192	-0.40 (-0.99 to 0.20)	11	-0.12 (0.07)	0.095	-0.51 (-1.1 to 0.09)	11	-0.11 (0.07)	0.151	-0.44 (-1.03 to 0.16)
**ADNI MCI patients**												
main	7	0.02 (0.07)	0.802	0.10 (-0.65 to 0.85)	7	-0.10 (0.08)	0.184	-0.51 (-1.26 to 0.24)	7	-0.13 (0.08)	0.110	-0.61 (-1.37 to 0.14)
unadjusted	7	0.02 (0.08)	0.798	0.10 (-0.65 to 0.85)	7	-0.08 (0.08)	0.325	-0.38 (-1.13 to 0.37)	7	-0.11 (0.09)	0.237	-0.45 (-1.20 to 0.30)
**Pooled MCI patients**												
main	18	-0.02 (0.04)	0.686	-0.10 (-0.56 to 0.37)	18	-0.12 (0.05)	0.015	-0.58 (-1.05 to -0.11)	18	-0.12 (0.05)	0.017	-0.57 (-1.04 to -0.10)
unadjusted	18	-0.04 (0.05)	0.415	-0.19 (-0.66 to 0.27)	18	-0.10 (0.05)	0.049	-0.47 (-0.93 to 0.00)	18	-0.11 (0.06)	0.063	-0.44 (-0.91 to 0.02)

Note. Main analyses were adjusted for age, gender, APOE-ε4 status and origin of the CSF samples. The unadjusted analyses included no additional covariates to p.P522R.
N(p.P522R)= Number of p.P522R carrier. Est= Estimate, SE= Standard error, P= p-value, d= standardized mean differences (Cohen's d), 95%-CI= 95% Confidence interval, tTau levels= total Tau levels in CSF, MCI= Mild cognitive impairment.

Supplementary Table 7. Interaction of p.P522R with CSF sample.

	$A\beta_{1-42}$ levels		$pTau_{181}$ levels		tTau levels	
**MCI patients**	**χ^2 (df)**	**p**	**χ^2 (df)**	**p**	**χ^2 (df)**	**p**
Interaction p.P522R x CSF sample	2.74 (2)	0.254	1.75 (2)	0.417	2.59 (2)	0.273
	**Est. (SE)**	**p**	**Est. (SE)**	**p**	**Est. (SE)**	**p**
p.P522R	0.03(0.07)	0.722	-0.11(0.08)	0.149	-0.14(0.08)	0.094
Bonn Sample	0.04(0.04)	0.329	-0.01(0.04)	0.797	0.03(0.05)	0.441
Erlangen sample	-0.03(0.01)	0.046	0.00(0.02)	0.88	0.03(0.02)	0.046
Amsterdam sample	-0.02(0.01)	0.106	0.00(0.02)	0.95	0.04(0.02)	0.015
p.P522R*Bonn Sample[a]	-	-	-	-	-	-
p.P522R*Erlangen Sample	-0.13(0.1)	0.186	-0.05(0.11)	0.665	-0.02(0.11)	0.883
p.P522R*Amsterdam Sample	0.07(0.15)	0.641	0.17(0.17)	0.312	0.25(0.17)	0.149

Note. Analyses were adjusted for age, gender, APOE-ε4 status.

a: The Bonn sample included no p.P522R carrier.

$A\beta_{1-42}$ levels=amyloid beta 1-42 levels in CSF, $pTau_{181}$ levels =phosphorylated tau levels in CSF, tTau levels= total Tau levels in CSF, Est== Estimate, SE= Standard error. P= p-value. d= standardized mean differences (Cohen's d), 95%-CI= 95% Confidence interval, tTau levels= total Tau levels in CSF, MCI= Mild cognitive impairment, df=degrees of freedom; χ^2=statistic of the multivariate Wald test.

Supplementary Table 8. Effect of p.P522R on CSF biomarkers of AD in comparison to the effect of APOE-ε4

Type of analysis	$A\beta_{1\text{-}42}$ levels				$pTau_{181}$ levels				tTau levels			
	N (c)	Est. (SE)	p	d (95%-CI)	N (c)	Est. (SE)	p	d (95%-CI)	N (c)[a]	Est. (SE)	p	d (95%-CI)
p.P522R	18	-0.02 (0.04)	0.686	-0.10 (-0.56 to 0.37)	18	-0.12 (0.05)	0.015	-0.58 (-1.05 to -0.11)	18	-0.12 (0.05)	0.017	-0.57 (-1.04 to -0.10)
APOE-ε4	593	-0.17 (0.01)	$1.36*10^{-52}$	-0.91 (-1.02 to -0.79)	593	0.12 (0.01)	$3.51*10^{-22}$	0.56 (0.45 to 0.67)	578	0.15 (0.01)	$2.29*10^{-29}$	0.67 (0.55 to 0.78)

Note. Analyses were adjusted for age, gender, APOE-ε4 status and origin of the CSF samples.
N(c)= Number of p.P522R or APOE-ε4 carrier, Est=Estimate, SE= Standard error, P= p-value. d= standardized mean differences (Cohen's d), 95%-CI= 95% Confidence interval, $A\beta_{1\text{-}42}$ levels=amyloid beta 1-42 levels in CSF, $pTau_{181}$ levels =phosphorylated tau levels in CSF, tTau levels= total Tau levels in CSF, MCI= Mild cognitive impairment.

Supplementary Table 9. Interaction of p.P522R with APOE- ε4

	$A\beta_{1\text{-}42}$ levels			$pTau_{181}$ levels			tTau levels		
	χ^2	df	p	χ^2	df	p	χ^2	df	p
Interaction p.P522R x APOE- ε4	1.25	1	0.264	0.06	1	0.811	1.77	1	0.184
	**N (p.P522R)**	**Est. (SE)**	**p**	**N (p.P522R)**	**Est. (SE)**	**p**	**N (p.P522R)**	**Est. (SE)**	**p**
p.P522R	18	-0.08 (0.07)	0.255	18	-0.11 (0.07)	0.149	18	-0.20 (0.08)	0.011
APOE- ε4	18	-0.17 (0.01)	$1.12*10^{-52}$	18	0.12 (0.01)	$5.33*10^{-22}$	18	0.15 (0.01)	$3.74*10^{-28}$
APOE- ε4*p.P522R	18	0.1 (0.09)	0.265	18	-0.02 (0.1)	0.811	18	0.14 (0.1)	0.184

Note. Analyses were adjusted for age, gender, APOE-ε4 status and origin of the CSF samples.
N(p.P522R)= Number of p.P522R carrier. Est=Estimate, SE= Standard error, P= p-value, $A\beta_{1\text{-}42}$ levels=amyloid beta 1-42 levels in CSF, $pTau_{181}$ levels =phosphorylated tau levels in CSF, tTau levels= total Tau levels in CSF, MCI= Mild cognitive impairment.

Supplementary Table 10. Association of p.P522R and APOE-ε4 with the categories of the ATN framework in a multinomial regression model

	AD pathologic change (A+T-)		non-AD pathologic change (A- T+ or N+)		normal AD biomarkers (A-T-N-)	
Patients per group (N(p.P522R))	129 (7)		279 (2)		323 (4)	
Patients per group (N(APOE-ε4))	129 (62)		279 (95)		323 (82)	
	**OR (95%-CI)**	**p**	**OR (95%-CI)**	**p**	**OR (95%-CI)**	**p**
main	6.28 (3.32-11.88)	0.004	0.93 (0.39-2.24)	0.938	1.81 (0.86-3.83)	0.425
unadjusted	5.93 (3.28-10.75)	0.003	0.75 (0.32-1.73)	0.728	1.3 (0.66-2.55)	0.700

Note. Patients with an AD (A+T+N+) were chosen as the reference category and ORs indicate the enrichment in the respective ATN category in comparison to patients with AD. Therefore an OR>1 indicates that the carrying p.P522R is positively associated with this category. The references group consisted of 522 individuals of which 5 were p.P522R carriers.
The main analysis was adjusted for age, gender, and APOE-ε4. The unadjusted analyses included no additional covariates to p.P522R.
A=amyloid beta 1-42. T= phosphorylated tau. N=total tau. N(p.P522R)= Number of p.P522R carrier. N(APOE-ε4)= Number of APOE-ε4 carrier. OR=Odds ratio. 95%-CI= 95% Confidence interval. p=p-value.

Supplementary Table 11. Test statistics for the smooth term in the varying-coefficient generalized additive model representing the effect of p.P522R on tau pathology or neurodegeneration conditional on Abeta$_{1-42}$ levels

		$pTau_{181}$ levels				tTau levels			
		**F**	**edf**	**Ref.df**	**p**	**F**	**edf**	**Ref.df**	**p**
main	MCI	4.389	2.0	2.0	0.013	3.929	2.0	2.0	0.020
unadjusted	MCI	4.068	2.0	2.0	0.017	3.741	2.0	2.0	0.024

Note. The main analyses were adjusted for age, gender, APOE-ε4 status and origin of the CSF samples. The unadjusted analyses included no additional covariates to p.P522R.
$Aβ_{1-42}$ levels=amyloid beta 1-42 levels in CSF, $pTau_{181}$ levels =phosphorylated tau levels in CSF, tTau levels= total Tau levels in CSF, F=F-value of the test statistic. Edf=effective degrees of freedom of the smooth term. Ref.df= nominator degrees of freedom to compute the p-value of the F-statistic. p: p-value. tTau levels: total Tau levels in CSF. MCI: Mild cognitive impairment.

Supplementary Table 12. Results from structural equation model of the relationship between p.P522R, cognitive decline, and CSF Abeta$_{1-42}$ levels as well as pTau$_{181}$ and total Tau levels in CSF

pTau$_{181}$ levels [a]				tTau levels [b]			
	Est	SE	p		Est	SE	p
Abeta$_{1-42}$ on p.P522R	0.086	0.078	0.271	Abeta$_{1-42}$ on p.P522R	0.086	0.078	0.271
pTau$_{181}$ on p.P522R	-0.225	0.105	0.033	tTau on p.P522R	-0.224	0.115	0.051
pTau$_{181}$ on Abeta$_{1-42}$	-0.341	0.035	$2.42*10^{-22}$	tTau on Abeta$_{1-42}$	-0.365	0.038	$1.04*10^{-21}$
I on p.P522R	-1.943	3.256	0.551	I on p.P522R	-2.458	3.249	0.449
S on p.P522R	2.399	5.749	0.676	S on p.P522R	2.067	5.651	0.715
Q on p.P522R	-0.254	1.426	0.859	Q on p.P522R	-0.162	1.423	0.910
I on Abeta$_{1-42}$	4.117	1.021	$5.53*10^{-5}$	I on Abeta$_{1-42}$	3.168	1.016	0.002
S on Abeta$_{1-42}$	3.965	1.254	0.002	S on Abeta$_{1-42}$	3.753	1.254	0.003
Q on Abeta$_{1-42}$	-0.338	0.315	0.283	Q on Abeta$_{1-42}$	-0.307	0.314	0.327
I on pTau$_{181}$	-2.553	0.916	0.005	I on tTau	-5.000	0.824	$1.30*10^{-9}$
S on pTau$_{181}$	-2.158	1.124	0.055	S on tTau	-2.528	1.029	0.014
Q on pTau$_{181}$	0.011	0.295	0.971	Q on tTau	0.090	0.259	0.728

Note. a: Model fit indices: RMSEA=0.017, CFI=0.996, SRMR=0.059, see supplementary text 5.4.
b: Model fit indices: RMSEA=0.016, CFI=0.996, SRMR=0.056, see supplementary text 5.4.
Est=Estimate, SE=Standard error, p=p-value. pTau$_{181}$ levels =phosphorylated tau levels in CSF, tTau levels= total Tau levels in CSF, MCI=Mild cognitive impairment. Analyses were adjusted for age, gender, APOE-ε4 status and origin of the CSF samples.

Supplementary Table 13. Estimation of an indirect effect of p.P522R ion the cognitive change in the normalized MMSE from baseline mediated by pTau$_{181}$ and total Tau levels in CSF

pTau$_{181}$ levels	Est	CI-	CI+	Total Tau levels	Est	CI-	CI+
1-year change on p.P522R	0.484	0.002	1.160	1-year change on p.P522R	0.546	-0.040	1.271
2-year change on p.P522R	0.963	0.067	2.142	2-year change on p.P522R	1.052	-0.080	2.317
3-year change on p.P522R	1.438	0.100	3.097	3-year change on p.P522R	1.517	-0.114	3.140
4-year change on p.P522R	1.908	0.124	4.110	4-year change on p.P522R	1.942	-0.132	4.069
**Aβ$_{1-42}$ levels**	**Est**	**CI-**	**CI+**	**Aβ$_{1-42}$ levels**	**Est**	**CI-**	**CI+**
1-year change on p.P522R	0.296	-0.213	0.967	1-year change on p.P522R	0.278	-0.192	0.903
2-year change on p.P522R	0.535	-0.374	1.737	2-year change on p.P522R	0.504	-0.347	1.582
3-year change on p.P522R	0.715	-0.502	2.265	3-year change on p.P522R	0.680	-0.469	2.108
4-year change on p.P522R	0.838	-0.538	2.625	4-year change on p.P522R	0.804	-0.551	2.610

Note. Aβ$_{1-42}$ levels=amyloid beta 1-42 levels in CSF, pTau$_{181}$ levels =phosphorylated tau levels in CSF, tTau levels= total Tau levels in CSF, Est=Estimate,CI+=upper bound of the bootstrap 95% confidence interval based on 1000 draws. CI-=lower bound of the bootstrap 95% confidence interval based on 1000 draws.

Supplementary Table 14. Interaction analyses between p.P522R and CSF biomarkers concerning the cognitive decline in the normalized MMSE

	$A\beta_{1-42}$ levels[a]				$pTau_{181}$ levels[b]				tTau levels[c]		
	**χ^2**	**df**	**p**		**χ^2**	**df**	**p**		**χ^2**	**df**	**p**
Wald test for interaction	8.856	2	0.012	Wald test for interaction	0.680	2	0.712	Wald test for interaction	0.290	2	0.865
	**Est**	**SE**	**p**		**Est**	**SE**	**p**		**Est**	**SE**	**p**
I on p.P522R	-0.625	3.669	0.865	I on p.P522R	-2.515	3.788	0.507	I on p.P522R	-2.530	3.899	0.516
S on p.P522R	0.813	4.548	0.858	S on p.P522R	2.367	4.780	0.621	S on p.P522R	0.533	5.010	0.915
Q on p.P522R	0.807	1.242	0.516	Q on p.P522R	-0.157	1.252	0.900	Q on p.P522R	0.067	1.313	0.960
I on $Abeta_{1-42}$	5.013	0.971	$2.44*10^{-7}$	I on $pTau_{181}$	-3.624	0.880	$3.82*10^{-5}$	I on tTau	-5.861	0.825	$1.24*10^{-12}$
S on $Abeta_{1-42}$	4.697	1.158	$4.99*10^{-5}$	S on $pTau_{181}$	-3.504	1.058	0.001	S on tTau	-3.565	1.003	$3.79*10^{-4}$
Q on $Abeta_{1-42}$	-0.316	0.297	0.287	Q on $pTau_{181}$	0.129	0.268	0.632	Q on tTau	0.161	0.260	0.535
I on p.P522R* $Abeta_{1-42}$	-9.112	11.902	0.444	I on p.P522R* $pTau_{181}$	-5.995	8.509	0.481	I on p.P522R* tTau	-0.816	7.182	0.910
S on p.P522R* $Abeta_{1-42}$	44.833	15.187	0.003	S on p.P522R* Tau_{181}	4.480	9.846	0.649	S on p.P522R* tTau	-3.725	8.880	0.675
Q on p.P522R* $Abeta_{1-42}$	-11.940	4.186	0.004	Q on p.P522R* $pTau_{181}$	-0.346	2.465	0.888	Q on p.P522R* tTau	0.545	2.242	0.808

Note. All analyses were adjusted for age, gender, education, APOE-ε4 and CSF sample. CSF biomarkers were analyzed in separate models.
a: model fit indices: RMSEA=0.018, CFI=0.994, SRMR=0.054, see supplementary text 5.4.
b: model fit indices: RMSEA=0.016, CFI=0.995, SRMR=0.058, see supplementary text 5.4.
c: model fit indices: RMSEA=0.018, CFI=0.994, SRMR=0.055, see supplementary text 5.4.
$A\beta_{1-42}$ levels=amyloid beta 1-42 levels in CSF, $pTau_{181}$ levels =phosphorylated tau levels in CSF, tTau levels= total Tau levels in CSF, N(p.P522R)=Number of p.P522R carrier. Est= Estimate, SE: Standard error. p: p-value. tTau levels: total Tau levels in CSF. df=degrees of freedom; χ^2=statistic of the multivariate Wald test.

Supplementary Figure 6. Plot of the interaction effect between p.P522R and Abeta1-42 levels in CSF concerning the cognitive decline over 4 years in the normalized MMSE.

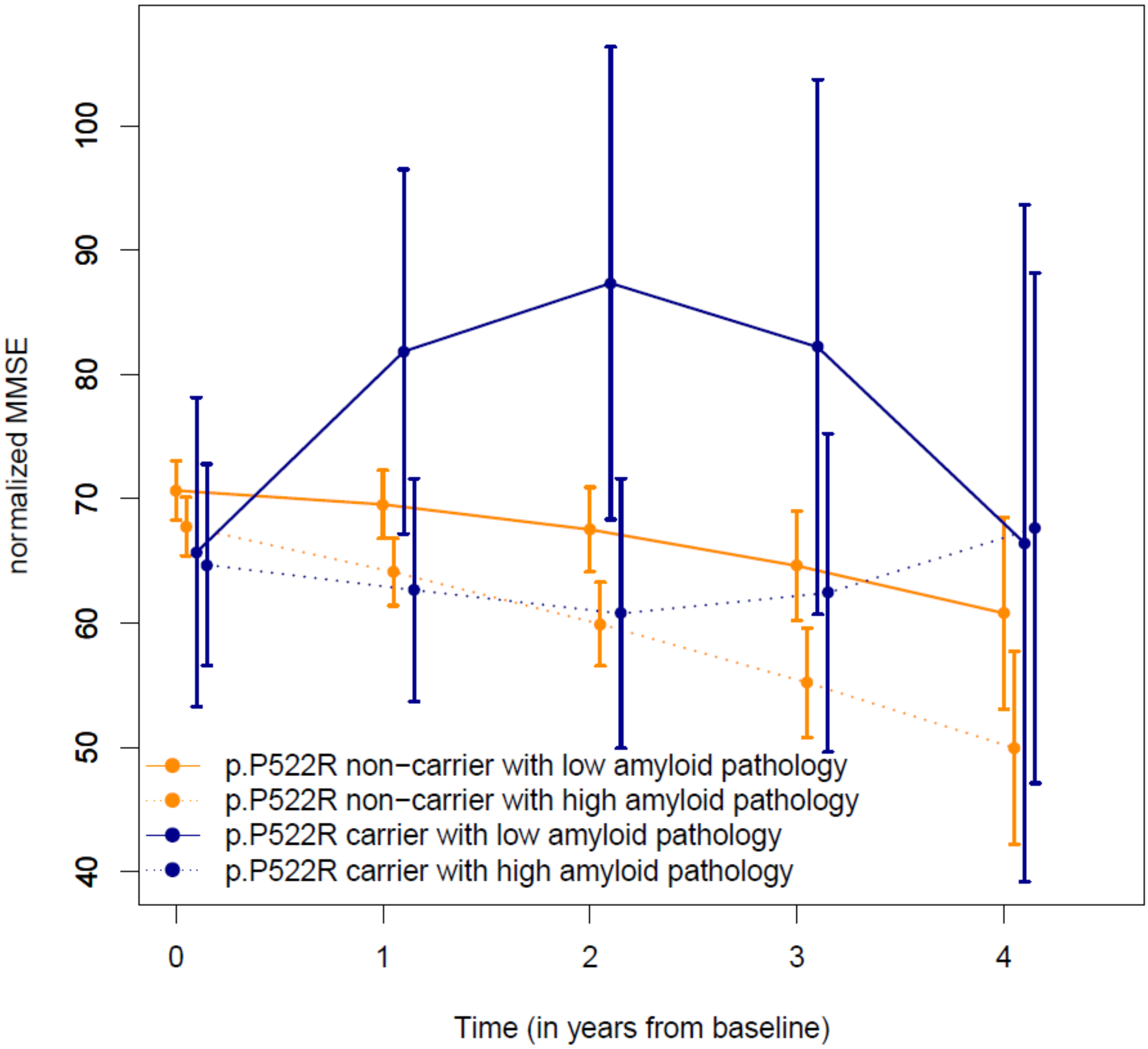

Note. The effect of p.P522R on the cognitive decline was evaluated at meaningful levels of Abeta$_{1\text{-}42}$ in CSF. For high amyloid pathology, we chose Abeta$_{1\text{-}42}$ CSF levels of 600pg/ml which is the cut-off for amyloid positivity in the CSF sample used as the reference (i.e. the DCN cohort) in the harmonization procedure for CSF measurements (see supplementary text 3.2). For low amyloid pathology, we selected one SD above the sample mean, which was 1070 pg/ml in this sample.

Supplementary Table 15. Enriched pathways in the co-regulation network of PLCG2

Gene Ontology (GO) biological process pathway ID	pathway description	Enrichment	FDR p-value
GO:0002250	adaptive immune response	3.3572	$<10^{-16}$
GO:0002449	lymphocyte-mediated immunity	3.2614	$<10^{-16}$
GO:0001906	cell killing	3.0954	$<10^{-16}$
GO:0002285	lymphocyte activation involved in immune response	3.0609	$<10^{-16}$
GO:0002440	production of molecular mediator of immune response	2.9131	$<10^{-16}$
GO:0001819	positive regulation of cytokine production	2.7451	$<10^{-16}$
GO:0002237	response to molecule of bacterial origin	2.7408	$<10^{-16}$
GO:0002521	leukocyte differentiation	2.6672	$<10^{-16}$
GO:0002446	neutrophil-mediated immunity	2.6400	$<10^{-16}$
GO:0001818	negative regulation of cytokine production	2.6366	$<10^{-16}$

*Note***.** FDR=false discovery rate.

Supplementary Table 16. Enriched pathways in the co-regulation network of APOE

Gene Ontology (GO) biological process pathway ID	pathway description	Enrichment	FDR p-value
GO:0050727	regulation of inflammatory response	7.6917	6.3474e-7
GO:0072376	protein activation cascade	18.109	6.3474e-7
GO:0002526	acute inflammatory response	12.020	0.0000027578
GO:0002576	platelet degranulation	13.118	0.0000055631
GO:0006959	humoral immune response	8.4142	0.000011671
GO:0051604	protein maturation	6.3237	0.00016886
GO:0010038	response to metal ion	5.1998	0.0025467
GO:0002697	regulation of immune effector process	4.8586	0.0039744
GO:0007272	ensheathment of neurons	9.3334	0.0041462
GO:0052547	regulation of peptidase activity	4.4285	0.0069136

*Note***.** FDR=false discovery rate.

Supplementary Table 17. Enriched pathways in the co-regulation network of TREM2

Gene Ontology (GO) biological process pathway ID	pathway description	Enrichment	FDR p-value
GO:0002683	negative regulation of immune system process	8.1190	0.0000038106
GO:0050727	regulation of inflammatory response	7.9166	0.000044576
GO:0002764	immune response-regulating signaling pathway	11.611	0.00011007
GO:0002526	acute inflammatory response	5.8925	0.00033348
GO:0036230	granulocyte activation	11.809	0.00033348
GO:0006959	humoral immune response	5.7158	0.00037289
GO:0050866	negative regulation of cell activation	8.5887	0.00045555
GO:0070661	leukocyte proliferation	6.4062	0.00094674
GO:0002694	regulation of leukocyte activation	7.3966	0.00094674
GO:0002697	regulation of immune effector process	5.4014	0.00094674

*Note***.** FDR=false discovery rate.

Supplementary Table 18. Enriched pathways in the shared co-regulation network of APOE, TREM2, and PLCG2

Gene Ontology (GO) biological process pathway ID	pathway description	Enrichment	FDR p-value
GO:0050727	regulation of inflammatory response	12.307	0.0026615
GO:0072376	**protein activation cascade**	32.193	0.0026615
GO:0002683	negative regulation of immune system process	10.680	0.0037252
GO:0002526	acute inflammatory response	19.232	0.0081362
GO:0048771	tissue remodeling	19.232	0.0081362
GO:0006959	**humoral immune response**	12.239	0.038857
GO:0001818	negative regulation of cytokine production	11.570	0.041260
GO:0002446	neutrophil-mediated immunity	7.4642	0.041338
GO:0036230	granulocyte activation	7.4045	0.041338
GO:0032102	negative regulation of response to external stimulus	9.4929	0.060951

*Note***.** FDR=false discovery rate. Boldly printed processes are Gene Ontology parent terms of complement activation.

Supplementary Table 19. The overlap between genes from the APOE-TREM2-PLCG2 shared gene set and genes differentially expressed in microglia under neurodegenerative conditions

Neurodegenerative condition	Overlapping genes
Human AD patients[33]	APOE, C1QA, C1QB, CD14, SPP1, TYROBP, VSIG4
5XFAD AD mouse model[22]	APOE, GPNMB, LPL, SPP1, TMEM176A, TREM2, TYROBP
hMAPT-P301S tauopathy mouse model[14]	APOE, C1QB, C3, GPNMB, LPL, SPP1

References

1. Albert MS, DeKosky ST, Dickson D et al. (2011) The diagnosis of mild cognitive impairment due to Alzheimer's disease: Recommendations from the National Institute on Aging-Alzheimer's Association workgroups on diagnostic guidelines for Alzheimer's disease. Alzheimer's & dementia 7(3): 270–279
2. Antoniak M, Pugliatti M, Hubbard R et al. (2003) Vascular factors and risk of dementia: design of the Three-City Study and baseline characteristics of the study population. Neuroepidemiology 22(6): 316–325
3. Bauer DJ, Cai L (2009) Consequences of unmodeled nonlinear effects in multilevel models. J Educ Behav Stat 34(1): 97–114
4. Benton A (1965) Manuel pour l'Application du Test de Rétention Visuelle: Applications Cliniques et Expérimentales. Paris: Centre de Psychologie Appliquée
5. Blessed G, Tomlinson BE, Roth M (1968) The association between quantitative measures of dementia and of senile change in the cerebral grey matter of elderly subjects. Br J Psychiatry 114(512): 797–811
6. Boada M, Cejudo JC, Tarraga L et al. (2002) Neuropsychiatric inventory questionnaire (NPI-Q): Spanish validation of an abridged form of the Neuropsychiatric Inventory (NPI). Neurología (Barcelona, Spain) 17(6): 317–323
7. Curran PJ, Hussong AM (2009) Integrative data analysis: The simultaneous analysis of multiple data sets. Psychol Methods 14(2): 81
8. Das S, Forer L, Schönherr S et al. (2016) Next-generation genotype imputation service and methods. Nat Genet 48(10): 1284
9. Dik MG, Deeg DJH, Bouter LM et al. (2000) Stroke and apolipoprotein E ε4 are independent risk factors for cognitive decline: a population-based study. Stroke 31(10): 2431–2436
10. Donohue MC, Gamst AC, Thomas RG et al. (2011) The relative efficiency of time-to-threshold and rate of change in longitudinal data. Contemp Clin Trials 32(5): 685–693
11. Ehli EA, Abdellaoui A, Fedko IO et al. (2016) A method to customize population-specific arrays for genome-wide association testing. Eur J Hum Genet 25: 267 EP -
12. Espinosa A, Alegret M, Valero S et al. (2013) A longitudinal follow-up of 550 mild cognitive impairment patients: evidence for large conversion to dementia rates and detection of major risk factors involved. J Alzheimers Dis 34(3): 769–780
13. Folstein MF, Folstein SE, McHugh PR (1975) "Mini-mental state": a practical method for grading the cognitive state of patients for the clinician. J Psychiatr Res 12(3): 189–198
14. Friedman BA, Srinivasan K, Ayalon G et al. (2018) Diverse brain myeloid expression profiles reveal distinct microglial activation states and aspects of Alzheimer's disease not evident in mouse models. Cell Rep 22(3): 832–847
15. Hindmarch I, Lehfeld H, Jongh P de et al. (1998) The Bayer activities of daily living scale (B-ADL). Dement Geriatr Cogn Disord 9(Suppl. 2): 20–26
16. Hoffman L, Hofer SM, Sliwinski MJ (2011) On the confounds among retest gains and age-cohort differences in the estimation of within-person change in longitudinal studies: A simulation study. Psychol Aging 26(4): 778–791. doi: 10.1037/a0023910
17. Hu Li-tze, Bentler PM (1999) Cutoff criteria for fit indexes in covariance structure analysis: Conventional criteria versus new alternatives. Struct Equ Modeling 6(1): 1–55
18. Huisman M, Poppelaars J, van der Horst, Marleen et al. (2011) Cohort profile: the longitudinal aging study Amsterdam. Int J Epidemiol 40(4): 868–876
19. Iacobucci D, Saldanha N, Deng X (2007) A meditation on mediation: Evidence that structural equations models perform better than regressions. Journal of Consumer psychology 17(2): 139–153
20. Isaacs B, Kennie AT (1973) The Set test as an aid to the detection of dementia in old people. Br J Psychiatry 123(575): 467–470
21. Karran E, Mercken M, Strooper B de (2011) The amyloid cascade hypothesis for Alzheimer's disease: an appraisal for the development of therapeutics. Nat Rev Drug Discov 10(9): 698
22. Keren-Shaul H, Spinrad A, Weiner A et al. (2017) A unique microglia type associated with restricting development of Alzheimer's disease. Cell 169(7): 1276-1290. e17
23. Kornhuber J, Schmidtke K, Frölich L et al. (2009) Early and differential diagnosis of dementia and mild cognitive impairment. Dement Geriatr Cogn Disord 27(5): 404–417

24. Lewczuk P, Esselmann H, Otto M et al. (2004) Neurochemical diagnosis of Alzheimer’s dementia by CSF Aβ42, Aβ42/Aβ40 ratio and total tau. Neurobiol Aging 25(3): 273–281
25. Lewczuk P, Kornhuber J, Wiltfang J (2006) The German Competence Net Dementias: standard operating procedures for the neurochemical dementia diagnostics. J Neural Transm 113(8): 1075–1080
26. Lopez OL, Jagust WJ, DeKosky ST et al. (2003) Prevalence and classification of mild cognitive impairment in the Cardiovascular Health Study Cognition Study: part 1. Arch Neurol 60(10): 1385–1389
27. Lopez OL, Kuller LH, Becker JT et al. (2007) Incidence of dementia in mild cognitive impairment in the cardiovascular health study cognition study. Arch Neurol 64(3): 416–420
28. Louwersheimer E, Wolfsgruber S, Espinosa A et al. (2016) Alzheimer’s disease risk variants modulate endophenotypes in mild cognitive impairment. Alzheimer Dement 12(8): 872–881
29. Luck T, Riedel-Heller SG, Kaduszkiewicz H et al. (2007) Mild Cognitive Impairment in General Practice: Age-Specific Prevalence and Correlate Results from the German Study on Ageing, Cognition and Dementia in Primary Care Patients (AgeCoDe). Dement Geriatr Cogn Disord 24(4): 307–316. doi: 10.1159/000108099
30. MacKinnon DP, Lockwood CM, Williams J (2004) Confidence limits for the indirect effect: Distribution of the product and resampling methods. Multivariate Behav Res 39(1): 99–128
31. Maldonado G, Greenland S (1993) Simulation study of confounder-selection strategies. Am J Epidemiol 138(11): 923–936
32. Martin Maechler, Peter Rousseeuw, Christophe Croux et al. (2017) robustbase: Basic Robust Statistics. http://robustbase.r-forge.r-project.org/
33. Mathys H, Davila-Velderrain J, Peng Z et al. (2019) Single-cell transcriptomic analysis of Alzheimer’s disease. Nature: 1
34. McCarthy S, Das S, Kretzschmar W et al. (2016) A reference panel of 64,976 haplotypes for genotype imputation. Nat Genet 48(10): 1279
35. McKhann G, Drachman D, Folstein M et al. (1984) Clinical diagnosis of Alzheimer’s disease Report of the NINCDS- ADRDA Work Group* under the auspices of Department of Health and Human Services Task Force on Alzheimer’s Disease. c 34(7): 939
36. Mefford J, Witte JS (2012) The covariate’s dilemma. PLoS Genet 8(11): e1003096
37. Morris JC (1993) The Clinical Dementia Rating (CDR): current version and scoring rules. Neurology 43 (11): 2412-2412-a
38. Morris JC, Heyman A, Mohs RC et al. (1989) The consortium to establish a registry for Alzheimer’s disease (CERAD): I. Clinical and neuropsychological assessment of Alzheimer’s disease. Neurology 39(9): 1159–1165.
39. Mulder C, Verwey NA, van der Flier, Wiesje M et al. (2010) Amyloid-β (1–42), total tau, and phosphorylated tau as cerebrospinal fluid biomarkers for the diagnosis of Alzheimer disease. Clin Chem 56(2): 248–253
40. Muthén, L.K. and Muthén, B.O. (1998-2012). Mplus User’s Guide. Seventh Edition. Los Angeles, CA: Muthén & Muthén
41. Naj AC, Jun G, Reitz C et al. (2014) Effects of multiple genetic loci on age at onset in late-onset Alzheimer disease: a genome-wide association study. JAMA Neurol 71(11): 1394–1404
42. Petersen RC, Smith GE, Waring SC et al. (1999) Mild cognitive impairment: clinical characterization and outcome. Arch Neurol 56(3): 303–308
43. Philipps V, Amieva H, Andrieu S et al. (2014) Normalized mini-mental state examination for assessing cognitive change in population-based brain aging studies. Neuroepidemiology 43(1): 15–25
44. Proust-Lima C, Dartigues J-F, Jacqmin-Gadda H (2011) Misuse of the linear mixed model when evaluating risk factors of cognitive decline. Am J Epidemiol 174(9): 1077–1088
45. Proust-Lima C, Philipps V, Liquet B (2017) Estimation of Extended Mixed Models Using Latent Classes and Latent Processes: The R Package lcmm. J Stat Softw 78(1): 1–56
46. Raghavan N, Samtani MN, Farnum M et al. (2013) The ADAS-Cog revisited: novel composite scales based on ADAS-Cog to improve efficiency in MCI and early AD trials. Alzheimer Dement 9(1): S21-S31
47. Rey A (1941) L’examen psychologique dans les cas d’encéphalopathie traumatique.(Les problems.). Archives de psychologie
48. Roberts R, Knopman DS (2013) Classification and epidemiology of MCI. Clin Geriatr Med 29(4): 10.1016/j.cger.2013.07.003-772. doi: 10.1016/j.cger.2013.07.003

49. Saan RJ, Deelman BG (1986) De 15-woordentest A en B (een voorlopige handleiding). Groningen: Afdeling Neuropsychologie, AZG
50. Shaw LM, Vanderstichele H, Knapik- Czajka M et al. (2009) Cerebrospinal fluid biomarker signature in Alzheimer's disease neuroimaging initiative subjects. Ann Neurol 65(4): 403–413
51. Shen L, Thompson PM, Potkin SG et al. (2014) Genetic analysis of quantitative phenotypes in AD and MCI: imaging, cognition and biomarkers. Brain Imaging Behav 8(2): 183–207
52. Sims R, Van Der Lee, Sven J, Naj AC et al. (2017) Rare coding variants in PLCG2, ABI3, and TREM2 implicate microglial-mediated innate immunity in Alzheimer's disease. Nat Genet 49(9): 1373
53. Szklarczyk D, Franceschini A, Wyder S et al. (2014) STRING v10: protein–protein interaction networks, integrated over the tree of life. Nucleic Acid Res 43(D1): D447-D452
54. van Dam S, Cordeiro R, Craig T et al. (2012) GeneFriends: an online co-expression analysis tool to identify novel gene targets for aging and complex diseases. BMC Genomics 13(1): 535
55. van der Flier, Wiesje M, Scheltens P (2018) Amsterdam Dementia Cohort: Performing Research to Optimize Care. J Alzheimers Dis 62(3): 1091–1111
56. Van Der Lee, Sven J, Conway OJ, Jansen I et al. (2019) A nonsynonymous mutation in PLCG2 reduces the risk of Alzheimer's disease, dementia with Lewy bodies and frontotemporal dementia, and increases the likelihood of longevity. Acta neuropathol 138: 1–14
57. van Rij J, Wieling M, Baayen RH et al. (2017) itsadug: Interpreting time series and autocorrelated data using GAMMs (published on the Comprehensive R Archive Network, CRAN)
58. Venables WN, Ripley BD (2013) Modern applied statistics with S-PLUS. Springer Science & Business Media
59. Weuve J, Proust-Lima C, Power MC et al. (2015) Guidelines for reporting methodological challenges and evaluating potential bias in dementia research. Alzheimer Dement 11(9): 1098–1109
60. Winblad B, Palmer K, Kivipelto M et al. (2004) Mild cognitive impairment–beyond controversies, towards a consensus: report of the International Working Group on Mild Cognitive Impairment. J Intern Med 256(3): 240–246
61. Wood SN (2003) Thin plate regression splines. J R Stat Soc Series B Stat Methodol 65(1): 95–114
62. Wood SN (2011) Fast stable restricted maximum likelihood and marginal likelihood estimation of semiparametric generalized linear models. J R Stat Soc Series B Stat Methodol 73(1): 3–36
63. Zaudig M, Mittelhammer J, Hiller W et al. (1991) SIDAM–A structured interview for the diagnosis of dementia of the Alzheimer type, multi-infarct dementia and dementias of other aetiology according to ICD-10 and DSM-III-R. Psychol Med 21(1): 225–236
64. Zhang B, Kirov S, Snoddy J (2005) WebGestalt: an integrated system for exploring gene sets in various biological contexts. Nucleic Acids Res 33(suppl_2): W741-W748
65. Zhou HH, Singh V, Johnson SC et al. (2018) Statistical tests and identifiability conditions for pooling and analyzing multisite datasets. Proc Natl Acad Sci: 115(7): 1481-1486
66. Zwan MD, Rinne JO, Hasselbalch SG et al. (2016) Use of amyloid-PET to determine cutpoints for CSF markers A multicenter study. Neurology 86(1): 50–58
